# Supramolecular conformational control of photolability in polymer networks crosslinked with a kinetically stable pseudo[1]rotaxane based on a coumarinylmethyl ester

**DOI:** 10.1039/d5sc01641j

**Published:** 2025-08-19

**Authors:** Hiroshi Masai, Naoki Niikura, Go M. Russell, Yutaro Kawano, Susumu Tsuda, Tomohiro Iwai, Jun Terao

**Affiliations:** a Department of Basic Science, Graduate School of Arts and Sciences, The University of Tokyo 3-8-1, Komaba Meguro-ku Tokyo 153-8902 Japan cmasai.h@g.ecc.u-tokyo.ac.jp cterao@g.ecc.u-tokyo.ac.jp; b PRESTO, Japan Science and Technology Agency 4-1-8, Honcho Kawaguchi Saitama 332-0012 Japan; c Department of Chemistry, Osaka Dental University 8-1 Kuzuhahanazonocho Hirakata Osaka 573-1121 Japan

## Abstract

Photolabile polymer materials have attracted considerable scientific and societal attention owing to the spatiotemporal control of light. However, easily photoreactive polymers are intrinsically unstable, leading to a tradeoff between their reactivity and stability. This article reports a proof-of-concept for reversible switching between photostable and photolabile states in polymer network materials enabled by the supramolecular transformation of the kinetically stable pseudo[1]rotaxane. A photocleavable coumarinylmethyl ester derivative, covalently linked with permethylated α-cyclodextrins, can be switched between insulated and uninsulated structures through conformational isomerization by altering the solvent polarity and heating. The hydrophobic environment of the cyclodextrin in the insulated structure partially inhibits polar solvation of the contact ion pair, an intermediate in the photocleavage reaction, thereby decreasing the photoreactivity of the coumarinylmethyl ester. Consequently, polymer network materials crosslinked with the insulated structure exhibit photostability, whereas the corresponding uninsulated structure is photolabile, demonstrating the reversible control of material photoreactivity *via* supramolecular conformational transformations.

## Abstract 2

A polymer network material crosslinked with the insulated coumarinylmethyl ester derivative enables control of the photolability *via* a quantitative and reversible intramolecular transformation between supramolecular conformational isomers.

## Graphical abstract

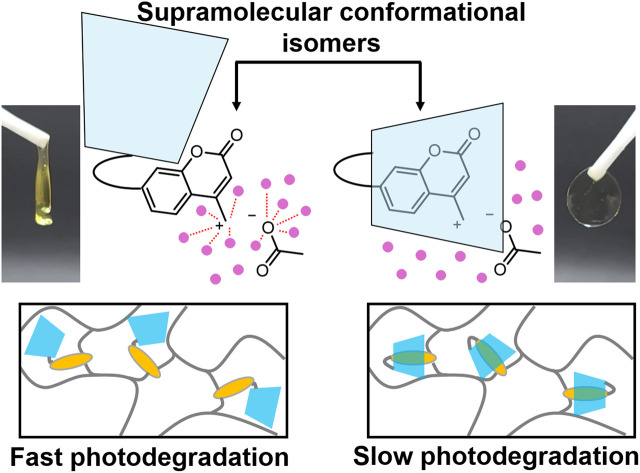

## Introduction

Light can induce macroscopic changes in photolabile materials such as deformation, softening, or liquefaction.^1–4^ The remote adaptability and high spatiotemporal control of light facilitate the precise tuning of the shape and physical or chemical properties of materials. As a result, photolabile materials have been utilized in various applications, including photoprocessing, soft actuators, and drug delivery systems.^5–7^ These materials can efficiently respond by cleaving the crosslinking points of the polymer network *via* external stimuli.^8,9^ Thus, photolabile polymers incorporating photocleavable crosslinkers, such as *o*-nitrobenzyl derivatives, coumarinylmethyl esters, and metal complexes, have been increasingly reported.^9–14^ Although these materials are typically designed to be highly sensitive to light for increased responsiveness, their rapid photoresponsiveness can also render them susceptible to light. This can lead to unintended decomposition under ordinary light conditions, restricting their long-term practical applications. Conversely, increasing the stability of materials to light can compromise their photoresponsiveness, presenting a trade-off between rapid photoreactivity and long-term photostability. However, overcoming these challenges through the on-demand active control of both the photoresponsive and photostable states could broaden the applications of these materials, rendering them suitable for long-term use under ordinary light conditions and enabling efficient photoreactivity under weak light conditions.

The active control of the photoreactive and photostable states in polymer materials has recently gained increasing attention as a compatible methodology between photostability and photoreactivity.^15–21^ For example, multistep and concerted reactions with light and additional stimuli in polymer materials have been explored for advanced photoresponsiveness beyond simple photodegradation. The new type of photoreactivity enables on-demand control of the photoreactivity and photostability of materials.^22^ Our research group recently discovered the acid-induced photodegradability of insulated platinum acetylide complexes with permethylated α-cyclodextrin (PM α-CD) which show concerted reactivity with light and acid, leading to the development of polymer network materials with photoresponsiveness in the presence of acid.^23,24^ The platinum complex demonstrated high photostability against acid owing to the steric effect of cyclodextrins. In contrast, when exposed to both light and acid, the complex exhibited cooperative cleavage reactivity. This unique property allows for novel reactivity in materials employing the complex as a network crosslinker.

**Fig. 1:**
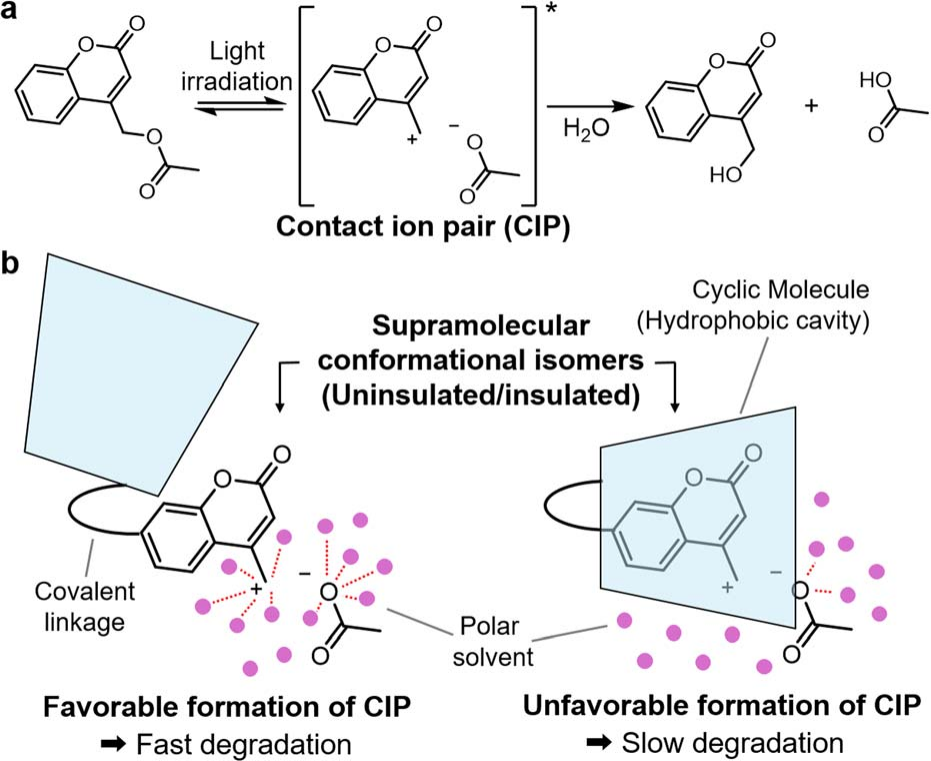
(a) Photocleavage reaction of coumarinylmethyl ester derivatives *via* a CIP intermediate. (b) Conceptual diagram illustrating the control of the photolability using uninsulated and insulated structures (supramolecular conformational isomers).

Herein, a new molecular design for controlling the photodegradability and photostability of materials was developed through the supramolecular conformational isomerization of the pseudo[1]rotaxane structure within polymer networks. Supramolecular host-guest structures are widely used to stabilize guest molecules through the steric hindrance provided by the inner cavity of cyclic molecules.^25–29^ In this study, a kinetically stable pseudo[1]rotaxane composed of photolabile guest moieties covalently linked with PM α-CD as a host was designed. The structure facilitated reversible switching between the insulated and uninsulated states through supramolecular conformational isomerization.^30–34^ Furthermore, by introducing appropriate steric barriers to threading and dethreading in pseudo[1]rotaxanes, each isomer was kinetically stabled at room temperature, while intramolecular isomerization was triggered by altering the solvent polarity and heating.^35^ As the photolabile guest moiety, coumarinylmethyl ester derivatives were used,^10,36–40^ which involves the formation of contact ion pairs (CIPs) upon light irradiation, as a proposed reaction mechanism of photodegradation (Fig. 1a).^41–43^ The CIP intermediates can be stabilized in polar solvents, which increases the photolabile reaction rate. In contrast, in the insulated structure, the inner cavity of PM α-CD provides a hydrophobic environment around the coumarinylmethyl ester moiety, resulting in slower reaction rates due to the inhibition of the formation of ionic species in the hydrophobic cavity (Fig. 1b). Thus, this methodology offers photoreactive and photostable states for photolabile polymer materials that are reversible *via* conformational control. Although several supramolecular structures have been employed to control photoreactivity,^44–47^ the reversible intramolecular switching of photolability and its application to the photoresponsiveness of polymer materials have not been reported. In this study, the photostability and photodegradability of polymer materials were actively controlled by the supramolecular isomerization of the kinetically stable pseudo[1]rotaxane comprising photolabile coumarinylmethyl ester derivatives covalently linked to PM α-CD. This strategy allows the materials to transition between a photostable state and a photolabile state on-demand.

## Results and discussion

**Scheme 1:**
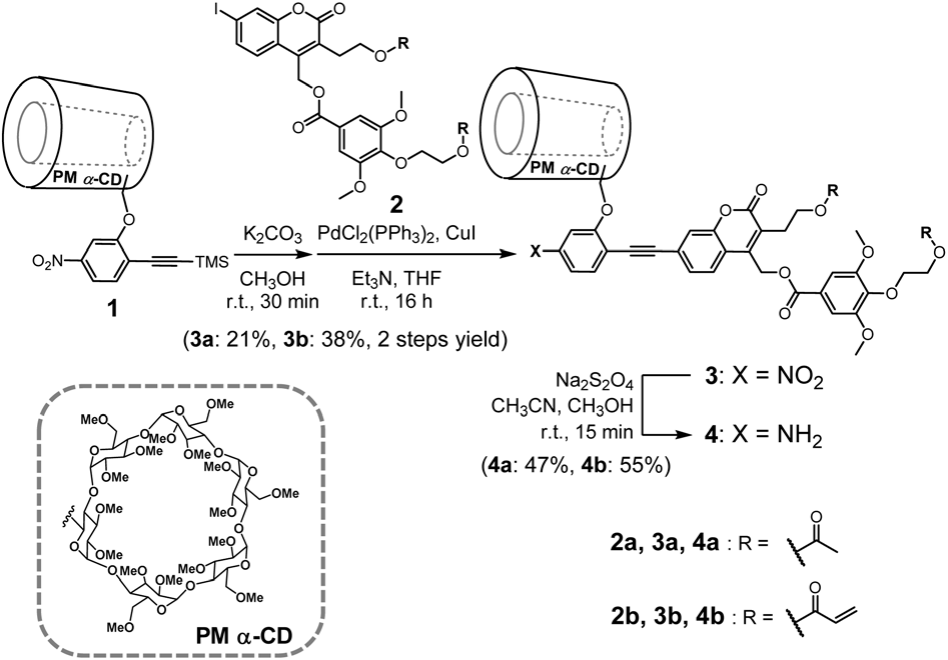
Synthesis route of the uninsulated coumarinylmethyl ester derivatives (4a and 4b).

Coumarinylmethyl ester derivatives bearing PM α-CD as the side chain (4a and 4b) were synthesized, as shown in Scheme 1.^48^ A Sonogashira coupling reaction was conducted between an ethynylbenzene derivative bearing PM α-CD (1) and a coumarinylmethyl ester bearing an iodo group (2), resulting in an uninsulated coumarinylmethyl ester derivative (3). The dimethoxybenzene moiety was introduced on 3 to monitor the photocleavage of the coumarinylmethyl ester using ^1^H NMR spectroscopy because the hydrogen atoms on the dimethoxybenzene moiety exhibited characteristic chemical shifts after photodegradation (∼7.2 ppm). Subsequently, the nitro groups on 3a and 3b were converted into the corresponding amino groups using sodium hydrosulfite, yielding 4a to investigate the photoreactivity and 4b for use as a crosslinking reagent.

**Fig. 2:**
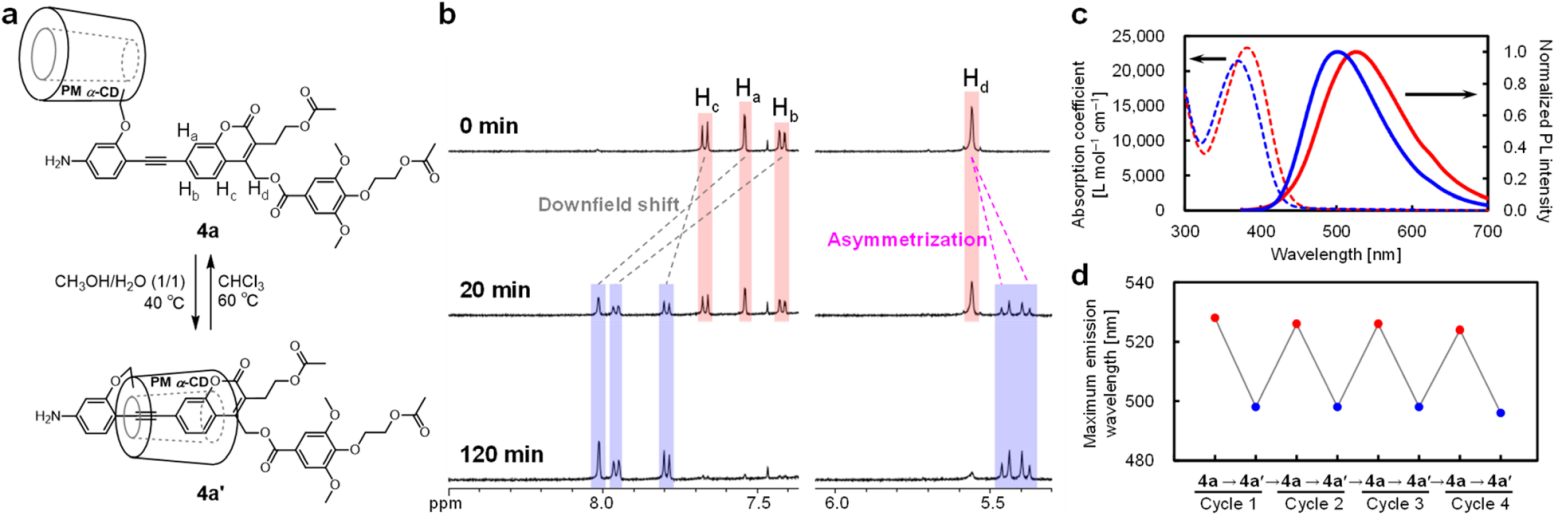
(a) Intramolecular conversion between supramolecular conformational isomers (4a and 4a′). (b) Time-course of the ^1^H NMR spectra (500 MHz, CDCl_3_, r.t.) from 4a to 4a′ under CH_3_OH–H_2_O (1/1, v/v) at 40 °C. (c) UV-vis absorption (dashed lines) and fluorescent (solid lines) spectra of 4a (red) and 4a′ (blue) in CHCl_3_. (d) Variations in the maximum fluorescent wavelength under repetitive transformation cycles between the insulated (4a′) and uninsulated (4a) structures (excitation at 365 nm in CHCl_3_).

Compound 4a underwent supramolecular conformational isomerization to its insulated counterpart (4a′) by heating the solution in CH_3_OH–H_2_O (1/1, v/v) at 40 °C, using a hydrophilic solvent mixture (Fig. 2a). ^1^H NMR analyses demonstrated the quantitative intramolecular transformation of 4a to 4a′ within 2 h, as shown in Fig. 2b (a Detailed discussion is provided in the SI). In addition, treating the insulated structure (4a′) in CHCl_3_ at 60 °C for 10 h quantitatively converted it back to its uninsulated counterpart (4a), demonstrating the reversible transformation between the insulated and uninsulated structures (Fig. S2). Notably, the amino group at the axle terminal significantly increases the activation barrier for the threading/dethreading of the pseudo[1]rotaxane owing to steric hindrance.^49^ As a result, the ^1^H NMR spectra indicated that 4a and 4a′ remained stable at r.t. after 6 h, even in thermodynamically unfavorable solvents, including CDCl_3_ for 4a′ and CD_3_OD for 4a (Fig. S3 and S4). Therefore, the supramolecular conformational isomers (4a and 4a′) were kinetically stable across a range of solvent polarities, enabling systematic investigation of insulation effects on photoreactivity by directly comparing insulated and uninsulated species in identical solvents.

The UV-vis absorption spectra of 4a and 4a′ in CHCl_3_ exhibit a broad absorption band at 320–450 nm, with maximum wavelengths of 382 and 372 nm, respectively (Fig. 2c). Furthermore, the emission spectra of 4a and 4a′ in CHCl_3_ under excitation at 365 nm exhibit maximum emission wavelengths at 525 and 501 nm, respectively, which is attributed to the donor–acceptor type emission of the coumarinylmethyl ester derivative.^48^ The blue-shift in the absorption and emission wavelengths of the insulated structure (4a′) was attributed to the twisted π-conjugated system resulting from the threading with PM α-CD, which decreased the effective conjugation length.^50^ Furthermore, reversible conformational isomerizations between 4a and 4a′ were achieved by heating solutions of 4a and 4a′ in CH_3_OH–H_2_O (1/1, v/v) and CHCl_3_, respectively. Notably, the maximum emission wavelengths remained consistent even after four transformation cycles (Fig. 2d). These optical measurements indicate the successful synthesis of coumarinylmethyl ester-based π-systems with insulated and uninsulated structures capable of repeated switching.

**Fig. 3:**
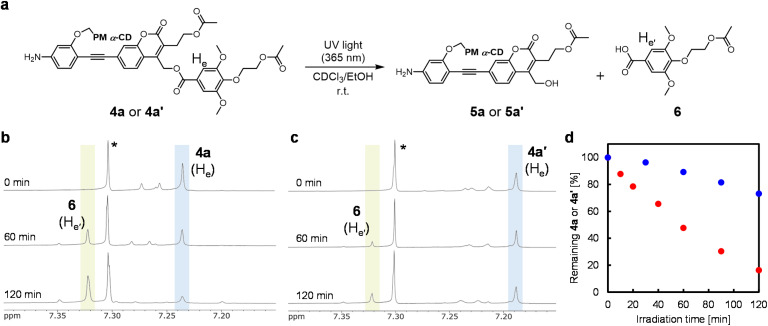
(a) Scheme of the photodegradation of 4a or 4a′ under 365 nm UV light. (b and c) Time-evolution of the ^1^H NMR spectra (500 MHz, CDCl_3_–EtOH (95/5, v/v), r.t.) of (b) 4a and (c) 4a′ before and after photoirradiation with 365 nm UV light (480 mW cm^−2^). Asterisk: CHCl_3_. (d) Time-course of photodegradation of 4a (red) or 4a′ (blue) analyzed by ^1^H NMR spectroscopy under 365 nm UV light irradiation.

The photodegradabilities of 4a and 4a′ were evaluated by tracing the ^1^H NMR spectra under light irradiation (Fig. 3a). Coumarinylmethyl ester derivative solutions (4a or 4a′) in CDCl_3_–EtOH (95/5, v/v) were exposed to 365 nm UV light (480 mW cm^−2^). Time-evolution of ^1^H NMR spectra indicated that 4a or 4a′ was converted during photoirradiation, generating the syringic acid derivative (6) as the degradation product (Fig. 3b, c, S5, and S6). Additionally, the MS measurement after the photoreaction of 4a′ supports the generation of coumarinylmethyl alcohol (5a or 5a′) and a syringic acid derivative (6) (Fig. S10). The ^1^H NMR analyses indicated that the degree of photodegradation for the uninsulated structure (4a) was approximately 70% after 90 min of exposure to 365 nm UV light (480 mW cm^−2^). In contrast, the photodegradation of the insulated structure (4a′) was significantly reduced to approximately 20% (Fig. 3d). Thus, the insulation of 4a′ effectively decreased the photodegradability of the coumarinylmethyl ester moiety, enhancing its photostability. Typical coumarinylmethyl esters exhibited increased photoreactivity in high-polarity solvents, indicating pronounced solvent dependence.^42^ Hence, the observed difference in photolability between 4a and 4a′ in CDCl_3_–EtOH was likely attributable to the low-polarity environment of the inner cavity within PM α-CD.

To reveal the effects of the supramolecular structures, the photocleavage reactivities of 4a and 4a′ were assessed using ^1^H NMR in DMSO-*d*_6_, a high-polarity solvent. The photodegradation degree of the uninsulated molecule (4a) increased 2.4-fold after 10 min in DMSO-*d*_6_ compared with that in the CDCl_3_–EtOH (95/5, v/v) mixture (Fig. S7 and S8). However, the photodegradation degree of 4a′ in DMSO-*d*_6_ was similar to that of 4a (Fig. S9), despite its insulated structure. This result is possibly due to the strong polar solvation in DMSO-*d*_6_, which may have surpassed the insulating effect on the coumarinylmethyl ester moiety. The CPK model of the insulated structure shown in Fig. S16 was obtained through computational calculations, revealing that the coumarinylmethyl ester was primarily located in the inner to peripheral region of PM α-CD, while the reactive site was slightly exposed to the external environment. Consequently, in the relatively low-polarity solvent (CDCl_3_–EtOH), the limited exposure was insufficient to stabilize the CIP intermediate, and the PM α-CD exerted an insulating effect. In contrast, in DMSO-*d*_6_, the high polarity solvent effectively stabilized even partially exposed ion-pair intermediates *via* solvation. This solvation effect would override the stabilizing effect of the macrocycle, resulting in similar photoreactivities of 4a and 4a′ in DMSO-*d*_6_. Although the stabilizing effects depended on the solvent system, this study reveals that isomeric supramolecular protection can govern intramolecular photoreactivity by modulating solvation dynamics at the reactive site. This highlights the potential of supramolecular conformational isomers to modulate the intramolecular photolability of polymer materials, suggesting a new strategy for controlling their durability and sensitivity under light exposure.

**Fig. 4:**
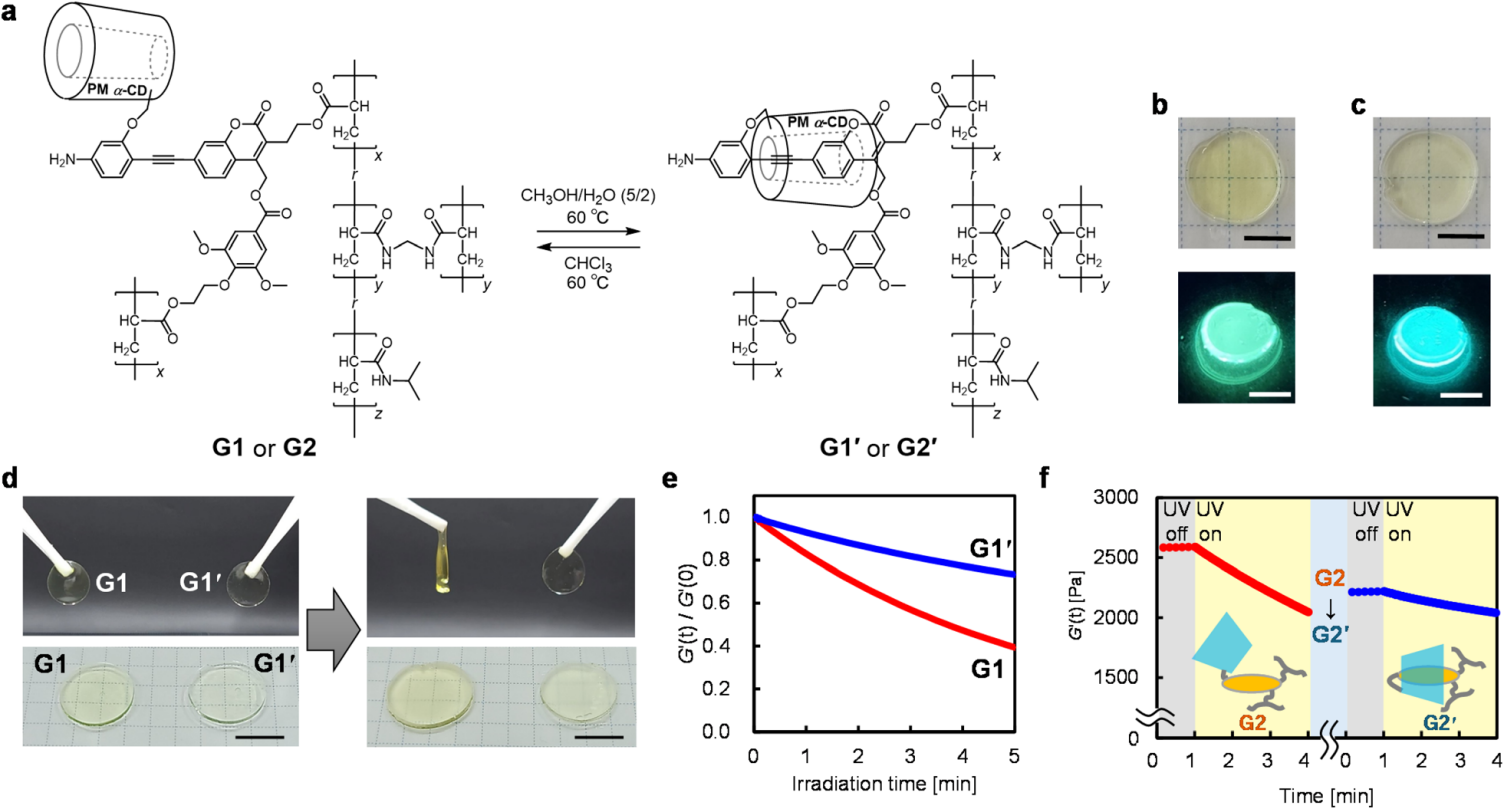
(a) Reversible transformations of the uninsulated and insulated gels. (b and c) Photographic images of (b) the G1 and (c) G1′ gels under white (top) and 365 nm UV light (bottom) irradiation (*x*/*y*/*z* = 0.15/0/100, scale bar: 5 mm). (d) Photographic images of the uninsulated (G1) and insulated (G1′) gels before (left) and after (right) photoirradiation (365 nm, 116 mW cm^−2^, scale bar: 10 mm). (e) Time-evolution of the storage moduli (*G*′) of photoirradiated (365 nm, 33 mW cm^−2^) G1 and G1′ in CHCl_3_–EtOH (95/5, v/v). (f) Time-evolution of the *G*′ of G2 (*x*/*y*/*z* = 0.1/0.1/100) and the gel subsequently transformed to an insulated structure (G2′) during photoirradiation (365 nm, 33 mW cm^−2^) measured in CHCl_3_–EtOH (95/5, v/v).

Polymer network materials incorporating coumarinylmethyl ester derivatives were synthesized *via* radical polymerization using *N*-isopropylacrylamide (NIPAM) as a monomer (1 eq.), 4b as a photolabile crosslinker (0.001 or 0.0015 eq.), and *N*,*N*′-methylenebisacrylamide (0 or 0.001 eq.) as a photostable crosslinker. The radical polymerization was initiated by 2,2′-azobis(2,4-dimethylvaleronitrile) (0.001 eq.) at 60 °C for 18 h in DMSO. The resulting network material (G1, *x*/*y*/*z* = 0.15/0/100 in Fig. 4a), which featured an uninsulated coumarinylmethyl ester moiety, yielded a transparent and elastic disc-shaped gel (Fig. 4b). G1, which is based on a poly(NIPAM), could retain solvents of various polarities. The swelling ratios, which are calculated based on the swollen mass to the dry mass of the gel, were 37, 47, and 12 for different polarity solvents (CHCl_3_, CHCl_3_–EtOH (95/5, v/v), and CH_3_OH–H_2_O (5/2, v/v), respectively). The result demonstrates the amphiphilic nature of the poly(NIPAM) network, which enhances its broad capability of retaining the polarity of solvents.

The uninsulated moiety in G1 was converted to its insulated counterpart (G1′) through supramolecular conformational isomerization by soaking it in a hydrophilic CH_3_OH–H_2_O (5/2, v/v) solution at 60 °C (Fig. 4a). The luminescence spectrum of the resulting gel exhibited a peak emission wavelength of 498 nm, which was blue-shifted by approximately 20 nm, as compared with that of the original gel, G1 (518 nm) (Fig. 4b, c, S11a, and S11e). This shift in the emission wavelength upon insulation corresponds with the observed trends in the conversion between compounds 4a and 4a′. Furthermore, the emission wavelengths of G1 and G1′ barely changed (∼3 nm) after 6 h even in high (CH_3_OH–H_2_O (5/2, v/v)) and low (CHCl_3_) polarity solvents, which are thermodynamically unfavorable solvents for the uninsulated and insulated structures, respectively (Fig. S11c, S11d, and S11f). The kinetic stability of the insulated structure was maintained even in the gel state (G1′). In contrast, under CHCl_3_ at 60 °C, the insulated structure of G1′ reverted to the uninsulated form, G1, and the emission spectrum shifted back to its original state (Fig. S11b and S11e). This reversible transformation between the insulated and uninsulated structures, along with their kinetic stabilization, was also demonstrated in the network material.

The macroscopic properties of the gel materials containing the insulated and uninsulated crosslinkers changed drastically due to the cleavage of the crosslinking points. G1 was degraded under photoirradiation and lost its original shape, whereas the corresponding insulated gel (G1′) maintained its original shape under the same irradiation conditions (365 nm, 116 mW cm^−2^, 20 min) (Fig. 4d). Even though the molar ratio of the photolabile crosslinker to the monomer was small (∼1/1000 eq.), the photoreactive crosslinker significantly influenced the macroscopic properties of the gel. Hence, the photoreactivity of the insulated and uninsulated coumarinylmethyl ester derivatives was reflected in the macroscopic photoresponsiveness of the polymer network material.

The photodegradabilities of the gels were quantitatively evaluated by tracing their dynamic viscoelasticity under 365 nm UV light irradiation. This process decreases the storage modulus (*G*′) of the gel, which is proportional to the crosslinking density. The *G*′ of the uninsulated gel (G1) decreased upon irradiation with 365 nm UV light (33 mW cm^−2^) using CHCl_3_–EtOH (95/5, v/v) as the retained solvent (Fig. 4e). Conversely, the *G*′ of the insulated gel (G1′) did not relatively decrease. These results indicate that the kinetically stable pseudo[1]rotaxane in the coumarinylmethyl ester enhances the photostability of the gels, which is consistent with the trends observed for 4a and 4a′. Moreover, the difference in the photodegradability between G1 and G1′ was dependent on the solvent polarity of the gels; The degradation rates of G1 and G1′ in a high-polarity solvent (DMSO) were approximately consistent (Fig. S12). The macroscopic photolabilities of G1 and G1′ successfully reflected the nanoscale molecular reactivities of 4a and 4a′.

Finally, the photoreactivity of the network can be switched after partial degradation by inducing a conformational transformation of the pseudo[1]rotaxane structure. G2 (*x*/*y*/*z* = 0.1/0.1/100), a polymer network material, was first irradiated with 365 nm UV light (33 mW cm^−2^) for 3 min in the retaining solvent CHCl_3_–EtOH (95/5, v/v). This cleaved a portion of the coumarinylmethyl-ester cross-links, lowering the normalized *G*′ by 21% (Fig. 4f). The partially photodegraded G2 was then insulated by solvent exchange to CH_3_OH–H_2_O (5/2, v/v) and heating to 60 °C, triggering isomerization to the insulated structure (G2′). After replacing the retaining solvent with CHCl_3_–EtOH (95/5, v/v), the material was re-irradiated under the same UV conditions, resulting in a slight decrease in normalized *G*′ by only 8% after 3 min. These results demonstrate that the conformational change of the kinetically stable pseudo[1]rotaxane converts the network from a photolabile to a photostable state, as intended. Accordingly, the internal molecular switch enables active control over the material's resistance to photodegradation.

## Conclusions

In summary, a photolabile coumarinylmethyl ester derivative was insulated by the kinetically stable pseudo[1]rotaxane using PM α-CD. This design enabled control of the photolability *via* a quantitative and reversible intramolecular transformation between supramolecular conformational isomers. The insulated structure enhanced photostabilization by creating a hydrophobic environment around the photolabile moiety, while the uninsulated counterpart exhibited efficient photodegradability. Furthermore, the adaptable photodegradability can be incorporated into polymers as crosslinking points, creating the first instance of photolabile polymer networks with reversible intramolecular switching of photoreactivity through supramolecular conformational isomerization. An examination of the photoreactivity of both the insulated and uninsulated molecules revealed that the polymer network with the insulated crosslinker remained stable under photoexcitation, whereas the uninsulated network exhibited photodegradability. Moreover, the photostability and photodegradability could be switched by the supramolecular conformational transformation of the kinetically stable pseudo[1]rotaxanes in the network materials. To advance this strategy from proof-of-concept to real-world application, several challenges remain, including the design of molecules suitable for efficient synthesis on an industrial scale and the development of simplified switching protocols compatible with practical materials. Despite these challenges, the present work establishes a new platform for on-demand control of material photolability, reconciling the long-standing trade-off between photostability and photoreactivity.

## Author contributions

H. M. and J. T. conceptualized the study, acquired funding, and administered the project. H. M., T. I., and J. T. supervised the project. G. M. R. conducted preliminary investigations. N. N., G. M. R., and Y. K. developed the methodologies and undertook the investigations, including the experiments and analyses. T. S. undertook the synthesis experiments. H. M. prepared the original manuscript draft, and all authors contributed to reviewing and editing the submitted version.

## Conflicts of interest

The authors declare no competing interests.

## Supplementary Material

SC-016-D5SC01641J-s001

## Data Availability

The SI includes experimental details and data from the NMR, MS, optical, and viscoelastic analyses. See DOI: https://doi.org/10.1039/d5sc01641j.
