# Supplementary material for: Supramolecular conformational control of photolability in polymer networks crosslinked with a kinetically stable pseudo[1]rotaxane based on a coumarinylmethyl ester
Source: Chem Sci. 2025 Aug 19;16(36):16737–43. doi: 10.1039/d5sc01641j (PMC12362465; doi:10.1039/d5sc01641j)
Supplement: SC-016-D5SC01641J-s001 [file SC-016-D5SC01641J-s001.pdf]

Supplementary Information (SI) for Chemical Science.
This journal is © The Royal Society of Chemistry 2025

Supplementary Information

Supramolecular conformational control of photolability in polymer networks crosslinked with kinetically stable pseudo[1]rotaxane based on coumarinylmethyl ester

Hiroshi Masai,[a,b]* Naoki Niikura,[a] Go M. Russell,[a] Yutaro Kawano,[a] Susumu Tsuda,[c] Tomohiro Iwai,[a] and Jun Terao[a]*

[a] *Department of Basic Science, Graduate School of Arts and Sciences, The University of Tokyo, 3-8-1, Komaba, Meguro-ku, Tokyo 153-8902, Japan.*

[b] *PRESTO, Japan Science and Technology Agency, 4-1-8, Honcho, Kawaguchi, Saitama 332-0012, Japan.*

[c] *Department of Chemistry, Osaka Dental University, 8-1 Kuzuhahanazonocho, Hirakata, Osaka 573-1121, Japan.*

Table of Contents

S1. General remarks S2
S2. Synthetic procedures S4
S2.1. Synthesis of coumarinylmethyl ester derivatives S4
S2.2. Synthesis of gels S14
S3. Supramolecular conformational isomerization of 4a and 4a' S16
S3.1. Experimental procedures S16
S3.2. Kinetic trace of 4a' S18
S3.3. Kinetic stability of 4a and 4a' S19
S4. Photodegradation measurements of 4a and 4a' S20
S4.1. Evaluation of photodegradation rate of 4a and 4a' S20
S4.2. Solvent dependency of photodegradation rate S21
S5. Identification of photodegraded product S23
S6. Measurements of swelling ratio of G1 and G1' S24
S7. Supramolecular conformational isomerization of G1 and G1' S24
S8. Kinetic trace of G1 and G1' S25
S9. Photodegradability of G1 and G1' S26
S10. Macroscopic photodegradation S28
S11. Computational calculations S29
S12. NMR spectra S31
S12.1. ^{1}H NMR and ^{13}C{^{1}H} NMR spectra S31
S12.2. ^{1}H-^{1}H ROESY NMR spectra S40
S13. References S41

S1. General remarks

Materials

All reagents were commercially obtained and used as received unless otherwise noted. **1**[1] and **S4**[2] were prepared according to the reported procedures. Degassed tetrahydrofuran (THF) was purchased from Kanto Chemical and further purified by passage through activated alumina under positive nitrogen pressure as described by Grubbs et al.[3] Dried potassium carbonate (K_2CO_3) was dried by heating under vacuum, before use.

Instruments

NMR Spectroscopy: ^{1}H NMR (500 MHz), ^{13}C{^{1}H} NMR (126 MHz) and ^{1}H-^{1}H ROESY NMR (500 MHz), were measured with a Bruker AVANCE-500 spectrometer. The ^{1}H NMR chemical shifts were reported relative to tetramethylsilane (TMS, 0.00 ppm) or residual protonated solvent in DMSO-d_6 (2.50 ppm). The ^{13}C{^{1}H} NMR chemical shifts were reported relative to $^{13}CDCl_3$ (77.16 ppm).

Mass Spectroscopy (MS): Electrospray ionization time-of-flight (ESI-TOF) mass spectra were recorded on micrOTOF II-KE02. For high-resolution (HR) analyses, NaTFA cluster ions were used as the internal mass calibration.

Preparative Recycling Gel Permeation Chromatography (GPC): Preparative recycling GPC was performed with a Shimadzu LC-20AP System equipped with Shodex K-4002L and Shodex K-4002.5L columns, a Shimadzu SPD-20A, and a Shimadzu RID-10A as the eluent at a flow rate of 14 ml min^{-1}, *or* a JAI LaboACE LC-5060 Plus II equipped with JAIGEL-2HH and 2.5HR columns, a UV-VIS 4ch 400HM using $CHCl_3$ as the eluent at a flow rate of 7.5 ml min^{-1}.

Absorption Spectroscopy: Ultraviolet-visible absorption spectra were measured at a concentration of 2.0×10^{-5} M with a Shimadzu UV-2600 model.

Luminescence Spectroscopy: Emission spectra were measured with a Shimadzu RF-6000 Spectro Fluorophotometer.

Rheology Measurement: Photorheological studies were carried out on a strain-controlled rheometer (Anton Paar, MCR302e), equipped with a spot-type UV source (Hamamatsu, LIGHTNINGCURE LC-L1, 365 nm). Disc-shaped samples (14 mm in diameter) were

placed on the top of a plate and fixed with a round plate (12 mm in diameter). As a standard setting, a frequency of 1 Hz and a shear strain of 1% were maintained during the measurement.

UV light irradiation: A spot UV lamp (CCS, HLV-24UV365-4WNRBT, 365 nm, 8 mm in diameter) was used unless otherwise noted.

S2. Synthetic procedures

S2.1. Synthesis of coumarinylmethyl ester derivatives

Synthesis of **S3**

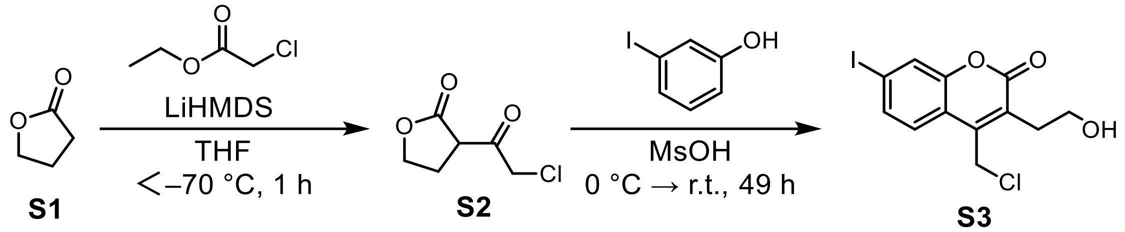

S1 **S2** **S3**

3.3% (2 steps)

Under a nitrogen atmosphere, LiHMDS solution (1 M in THF, 100 ml, 100 mmol) was added to degassed THF (100 ml). **S1** (9.10 ml, 120 mmol) was added dropwise to the solution of LiHMDS in THF at <–70 °C. After 20 min, ethyl chloroacetate (11.3 ml, 99.7 mmol) was added dropwise to the solution at <–70 °C. The reaction mixture was stirred for 1 h at <–70 °C. After 2 M HCl (163 ml) was added to the reaction mixture, the reaction mixture was further stirred for 10 min at 0 °C. The reaction mixture was then washed with brine and extracted with EtOAc. The combined organic layers were dried using $MgSO_4$, filtered, and concentrated. Thus-formed dark orange oil **S2** (14.7 g) was used without purification. Under a nitrogen atmosphere, 3-iodophenol (17.8 g, 80.9 mmol) was dissolved to methanesulfonic acid (97 ml). The crude product of **S2** (14.7 g) was added dropwise to the solution at 0 °C. The reaction mixture was allowed to warm to room temperature and stirred for 49 h. The reaction mixture was quenched with $NaHCO_3$ (sat. aq.) at 0 °C, and then extracted with CH_2Cl_2. The combined organic layers were dried using $MgSO_4$, filtered, and concentrated *in vacuo*. The residue was purified by column chromatography on silica gel (CH_2Cl_2:EtOAc = 20:0 to 20:1 to 4:1) to yield **S3** as a white solid (1.19 g, 3.27 mmol, 2 steps 3.3%).

ESI HR-MS: (m/z) 386.92724 ($[M+Na]^+$, $C_{12}H_{10}ClIO_3Na$, calcd. 386.92554).

^1H NMR (500 MHz, $CDCl_3$): δ_H = 7.75 (d, *J* = 1.6 Hz, 1H), 7.71-7.69 (m, 1H), 7.45 (d, *J* = 8.5 Hz, 1H), 4.79 (s, 2H), 3.96 (q, *J* = 5.7 Hz, 2H), 3.02 (t, *J* = 6.0 Hz, 2H), 1.71 (t, *J* = 5.1 Hz, 1H).

$^{13}C\{^1H\}$ NMR (126 MHz, $CDCl_3$): δ_C = 161.0, 152.6, 145.4, 133.8, 126.33, 126.17, 125.6, 117.5, 96.5, 60.9, 37.1, 30.8.

Synthesis of **S5**

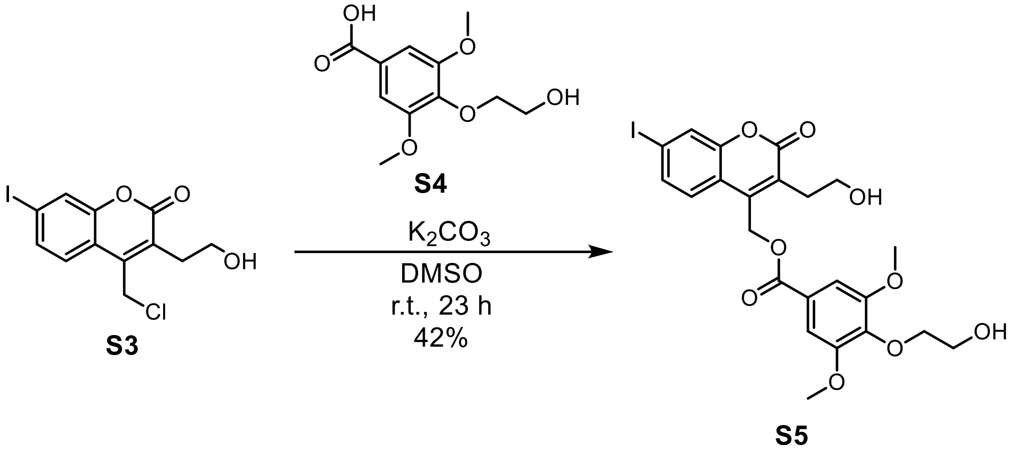

Under a nitrogen atmosphere, compound **S4** (517 mg, 2.13 mmol) and dried K_2CO_3 (829 mg, 6.00 mmol) were added into dry DMSO (22.5 ml). After 30 min stirring at room temperature, **S3** (725 mg, 1.99 mmol) was added to the solution. The reaction mixture was stirred in dark for 23 h at room temperature. The reaction mixture was then quenched with brine. The mixture was extracted with EtOAc. The combined organic layers were dried using $MgSO_4$, filtered, and concentrated *in vacuo*. The residue was purified by column chromatography on silica gel (CH_2Cl_2:EtOAc = 1:1) and GPC with $CHCl_3$ as the eluent to yield **S5** as a white solid. (474 mg, 0.830 mmol, 42%). Although the product contained impurities, it was used in the next step without further purification.

ESI HR-MS: (m/z) 593.02962 ($[M+Na]^+$, $C_{23}H_{23}IO_9Na$, calcd. 593.02790).

^1H NMR (500 MHz, $CDCl_3$): δ_H = 7.73 (s, 1H), 7.65 (d, J = 8.5 Hz, 1H), 7.53 (d, J = 8.5 Hz, 1H), 7.25 (s, 2H), 5.61 (s, 2H), 4.19 (t, J = 4.1 Hz, 2H), 3.94 (s, 2H), 3.89 (s, 6H), 3.74 (d, J = 3.1 Hz, 2H), 3.15 (t, J = 5.9 Hz, 2H).

$^{13}C\{^1H\}$ NMR (126 MHz, $CDCl_3$, r.t.): δ_C = 165.5, 161.1, 153.1, 152.5, 143.7, 141.2, 133.6, 127.9, 126.12, 125.99, 124.4, 118.4, 106.9, 96.3, 75.5, 61.4, 61.1, 59.5, 56.3, 31.0.

Synthesis of **2a**

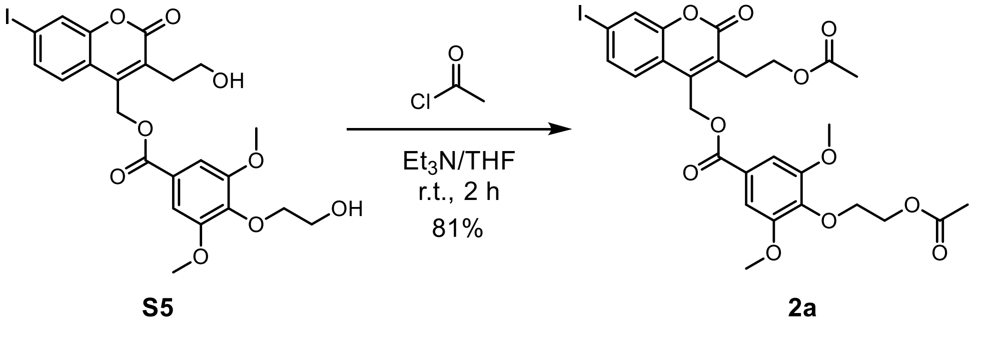

Under a nitrogen atmosphere, **S5** (474 mg, 0.831 mmol), and Et_3N (1.13 ml, 8.10 mmol) were dissolved into degassed THF (7.0 ml). After 10 min stirring at 0 °C, acetyl chloride (600 μl, 8.41 mmol) was added dropwise to the solution. The reaction mixture was allowed to warm to room temperature and stirred for 2 h. The reaction mixture was then washed with brine. The aqueous layer was extracted with CH_2Cl_2. The combined organic layers were dried using $MgSO_4$, filtered, and concentrated *in vacuo*. The residue was purified by column chromatography on silica gel (hexane:EtOAc = 1:1) and GPC with $CHCl_3$ as the eluent to yield **2a** as a white solid (438 mg, 0.670 mmol, 81%). Although the product contained impurities, it was used in the next step without further purification.
ESI HR-MS: (m/z) 677.04763 ($[M+Na]^+$, $C_{27}H_{27}IO_{11}Na$, calcd. 677.04903).
^{1}H NMR (500 MHz, $CDCl_3$, r.t.): δ_H = 7.74 (d, J = 1.4 Hz, 1H), 7.64 (dd, J = 8.5, 1.4 Hz, 1H), 7.49 (d, J = 8.5 Hz, 1H), 7.21 (s, 2H), 5.54 (s, 2H), 4.33 (dt, J = 10.1, 5.2 Hz, 4H), 4.26 (dd, J = 5.6, 3.8 Hz, 2H), 3.84 (s, 6H), 3.21 (t, J = 6.5 Hz, 2H), 2.07 (s, 3H), 2.00 (s, 3H).
$^{13}C\{^1H\}$ NMR (126 MHz, $CDCl_3$, r.t.): δ_C = 170.8, 170.6, 165.4, 160.3, 153.1, 152.5, 143.8, 141.5, 133.6, 126.8, 126.1, 124.1, 118.2, 107.0, 96.5, 70.8, 63.5, 62.4, 59.2, 56.3, 27.3, 20.8.

Synthesis of **3a**

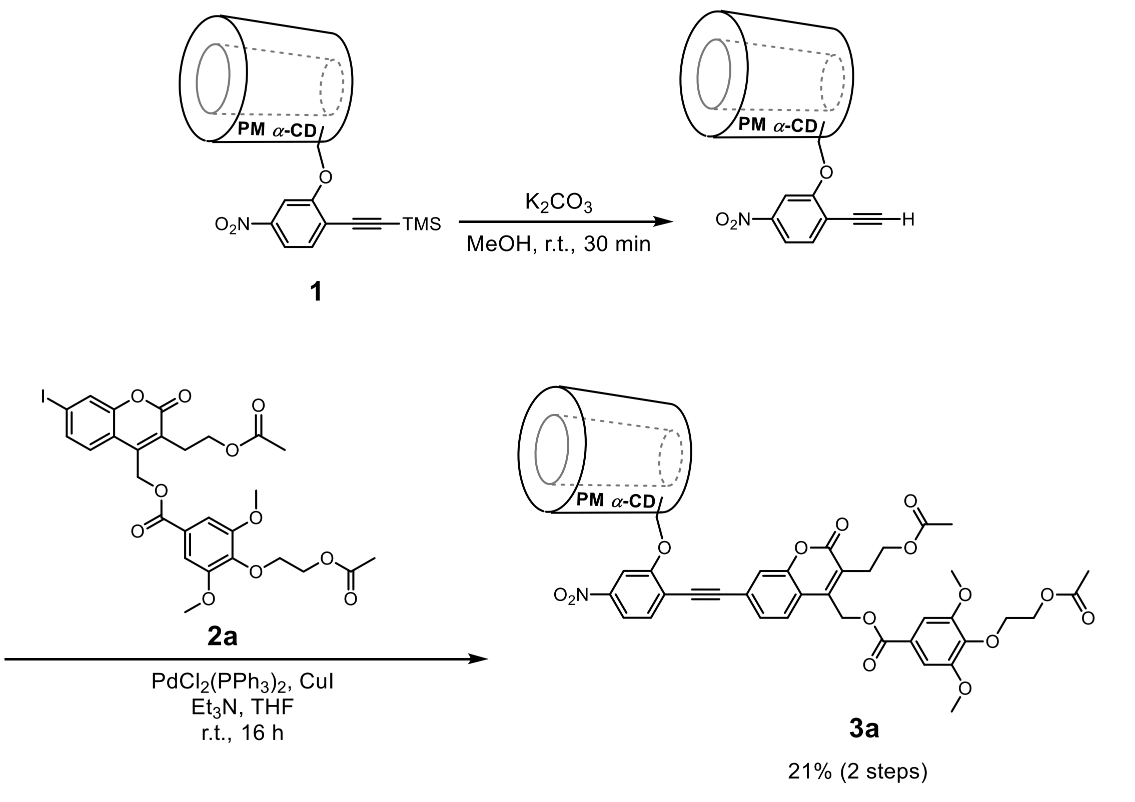

1 (367 mg, 0.257 mmol) and K_2CO_3 (107 mg, 0.770 mmol) were dissolved in MeOH (8.4 ml) under a nitrogen atmosphere. The reaction mixture was stirred at room temperature. After 30 min stirring, the mixture was dried *in vacuo* and then filtered through a Celite pad eluting with $CHCl_3$. The filtrate was concentrated, and the crude product was used immediately without further purification. The crude product was added into degassed THF (16.9 ml) and Et_3N (5.8 ml) and then stirred under nitrogen bubbling for 10 min. Under a nitrogen atmosphere, **2a** (168 mg, 0.257 mmol), CuI (2.4 mg, 13 μmol), and $PdCl_2(PPh_3)_2$ (8.8 mg, 13 μmol) were added into the solution, and then the reaction mixture was stirred at room temperature for 16 h. The reaction mixture was then washed with NH_4Cl (aq.) solution. The aqueous layer was extracted with EtOAc. The combined organic layers were dried using $MgSO_4$, filtered, and concentrated *in vacuo*. The residue was further purified by GPC with $CHCl_3$ as the eluent to yield **3a** as a yellow solid (99.7 mg, 52.9 μmol, 21%). Although the product contained impurities, it was used in the next step without further purification.
ESI HR-MS: (m/z) 1904.73655 ($[M+Na]^+$, $C_{88}H_{123}NO_{43}Na$, calcd. 1904.73610).
^1H NMR (500 MHz, $CDCl_3$, r.t.): δ_H = 7.87-7.85 (d and s, 1H), 7.82 (d, *J* = 1.9 Hz, 1H), 7.77 (d, *J* = 8.4 Hz, 1H), 7.64 (d, *J* = 6.2 Hz, 2H), 7.53 (dd, *J* = 8.3, 1.4 Hz, 1H), 7.24 (s, 2H), 5.59 (s, 2H), 5.08-3.03 (m, 107H), 2.08 (s, 3H), 2.02 (s, 3H).
$^{13}C\{^1H\}$ NMR (126 MHz, $CDCl_3$, r.t.): δ_C = 170.9, 170.7, 165.5, 160.5, 159.6, 153.2 (several peaks overlapped), 152.6, 148.3, 143.5, 141.5, 133.6, 127.7, 127.2, 125.5, 125.0, 124.1, 120.1, 119.22, 119.03, 115.9, 107.02 (several peaks overlapped), 106.88, 100.39, 100.28, 100.18, 100.14, 100.10, 96.5, 87.6, 82.69, 82.56, 82.52 (several peaks overlapped), 82.21, 82.18, 82.14 (several peaks overlapped), 82.02, 81.95, 81.18 (several peaks overlapped), 81.12 (several peaks overlapped), 81.02, 71.71, 71.67, 71.55, 71.52, 71.50, 71.45, 71.31, 71.29, 71.26, 71.18, 70.84 (several peaks overlapped), 70.14, 68.41, 63.55 (several peaks overlapped), 62.58, 61.81 (several peaks overlapped), 61.79 (several peaks overlapped), 61.75 (several peaks overlapped), 59.29, 59.19, 59.09 (several peaks overlapped), 59.08 (several peaks overlapped), 58.9, 58.2, 57.91, 57.87, 57.81, 57.79, 57.5, 56.30 (several peaks overlapped), 56.25, 27.4, 20.9 (several peaks overlapped).

Synthesis of **4a**

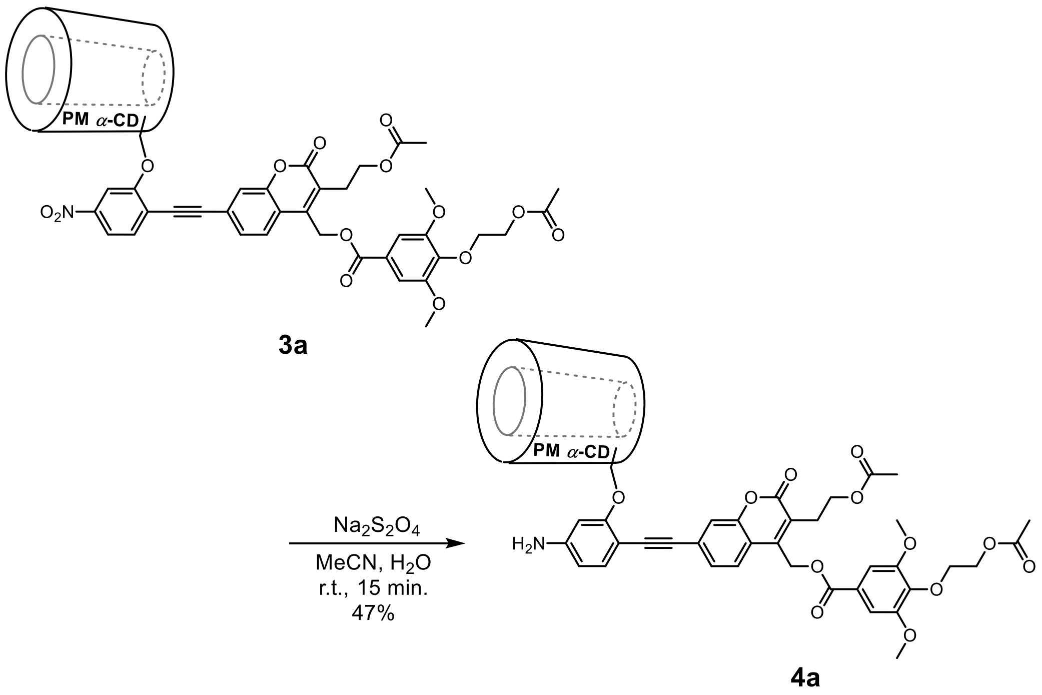

Under a nitrogen atmosphere, compound **3a** (99.7 mg, 52.9 µmol) and $Na_2S_2O_4$ (101.9 mg, 0.585 mmol) were dissolved into H_2O/MeCN (14.3/14.3 ml). After 15 min, the reaction mixture was diluted with brine. The aqueous layer was extracted with EtOAc. The combined organic layers were dried using $MgSO_4$, filtered, and concentrated. The residue was further purified by GPC with $CHCl_3$ as the eluent to yield **4a** as a yellow solid (45.9 mg, 24.8 µmol, 47%).

ESI HR-MS: (m/z) 1874.76270 ($[M+Na]^+$, $C_{88}H_{125}NO_{41}Na$, calcd. 1874.76192).

^1H NMR (500 MHz, $CDCl_3$): δ_H = 7.67 (d, J = 8.4 Hz, 1H), 7.54 (d, J = 1.5 Hz, 1H), 7.42 (dd, J = 8.4, 1.6 Hz, 1H), 7.28 (s, 1H), 7.23 (s, 2H), 6.25 (dd, J = 8.2, 2.0 Hz, 1H), 6.21 (d, J = 2.0 Hz, 1H), 5.56 (s, 2H), 5.13-3.06 (m, 109H), 2.07 (s, 3H), 2.01 (s, 3H).

^{13}C NMR (126 MHz, $CDCl_3$, r.t.): δ_C = 171.0, 170.7, 165.5, 160.97, 160.92, 153.16, 153.13, 152.6, 149.1, 143.8, 141.5, 134.6, 127.8, 127.3, 125.8, 124.6, 124.3, 119.1, 117.7, 107.6, 107.03, 106.98 (several peaks overlapped), 101.9, 100.34, 100.30, 100.21, 100.11, 100.06, 99.8, 99.2, 91.5, 90.5, 82.9, 82.57, 82.52 (several peaks overlapped), 82.38, 82.35, 82.27, 82.21 (several peaks overlapped), 82.19 (several peaks overlapped), 82.12, 82.03, 81.23 (several peaks overlapped), 81.18 (several peaks overlapped), 81.14, 81.12, 71.9, 71.51 (several peaks overlapped), 71.44, 71.39, 71.30 (several peaks overlapped), 71.25, 70.9 (several peaks overlapped), 70.2, 67.9, 63.6, 62.7, 61.87, 61.80, 61.78 (several peaks overlapped), 61.72, 61.70, 59.30, 59.16, 59.06, 59.05, 58.99, 58.89, 58.2, 57.93, 57.91, 57.85, 57.81, 57.5, 56.3 (several peaks overlapped), 27.2, 20.9 (several peaks overlapped).

Synthesis of **4a'**

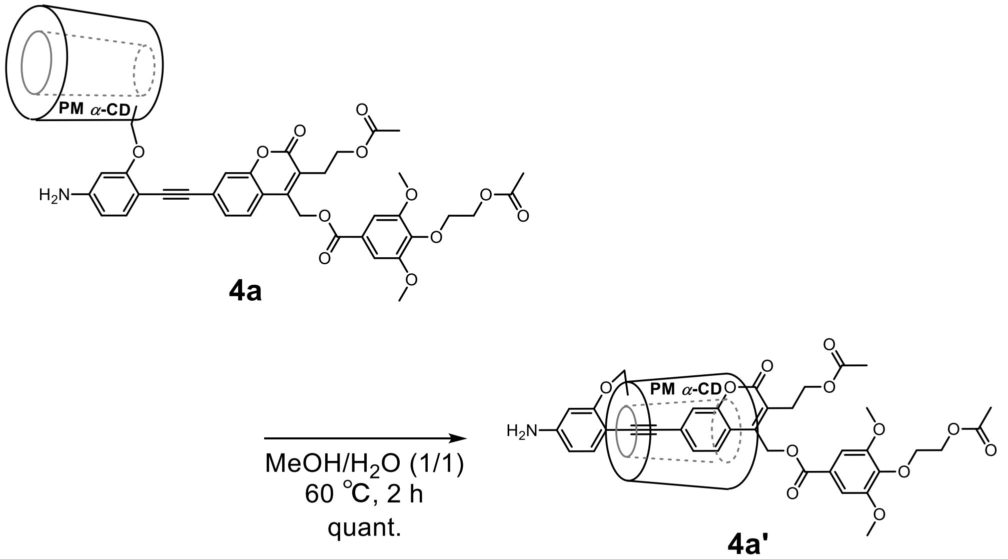

4a (5.4 mg, 2.9 μmol) was dissolved in MeOH (1 ml) and H_2O (1 ml). Under a nitrogen atmosphere, the mixture was stirred at 60 °C for 2 h. The mixture was then dried *in vacuo*, and the residue was diluted with water. The organic layer was extracted with EtOAc. The combined organic layers were dried over $MgSO_4$, filtered, and concentrated. The residue was further purified by GPC with $CHCl_3$ as the eluent to yield **4a'** as a yellow solid (3.1 mg, 57%.).

^1H NMR (500 MHz; $CDCl_3$): δ_H = 8.01 (d, *J* = 1.4 Hz, 1H), 7.95 (dd, *J* = 8.4, 1.4 Hz, 1H), 7.79 (d, *J* = 8.4 Hz, 1H), 7.24 (d, *J* = 8.5 Hz, 1H), 7.18 (s, 2H), 6.45-6.43 (m, 2H), 5.45 (d, *J* = 12.3 Hz, 1H), 5.39 (d, *J* = 12.3 Hz, 1H), 5.02 -2.85 (m, 109H), 2.08 (s, 3H), 2.00 (s, 3H).

^{13}C NMR (126 MHz, DMSO-d_6, r.t.): δ_C = 170.71, 170.61, 165.4, 163.8, 160.3, 153.1 (several peaks overlapped), 152.43, 152.26, 143.4, 141.3, 134.5, 128.6, 127.4, 127.1, 124.3, 123.8, 119.5, 118.1, 109.4, 107.0 (several peaks overlapped), 106.5, 101.7, 99.59, 99.41, 99.31, 99.24, 97.7, 93.1, 89.5, 83.1, 82.48 (several peaks overlapped), 82.32, 82.27, 82.23, 82.12, 82.05, 82.01, 81.91, 81.62, 81.43, 81.38 (several peaks overlapped), 81.21, 81.17, 81.12, 80.98, 79.6, 75.8, 72.7, 71.9, 71.46, 71.31, 71.22, 71.11, 71.09, 70.89 (several peaks overlapped), 70.82, 70.2, 63.6 (several peaks overlapped), 63.4, 61.63, 61.46, 61.32, 61.29, 61.0 (several peaks overlapped), 59.1, 58.85, 58.75, 58.68, 58.33, 58.32, 57.73, 57.64, 57.56, 57.37, 57.34, 57.32, 56.4 (several peaks overlapped), 26.7, 21.0, 20.8.

Synthesis of **2b**

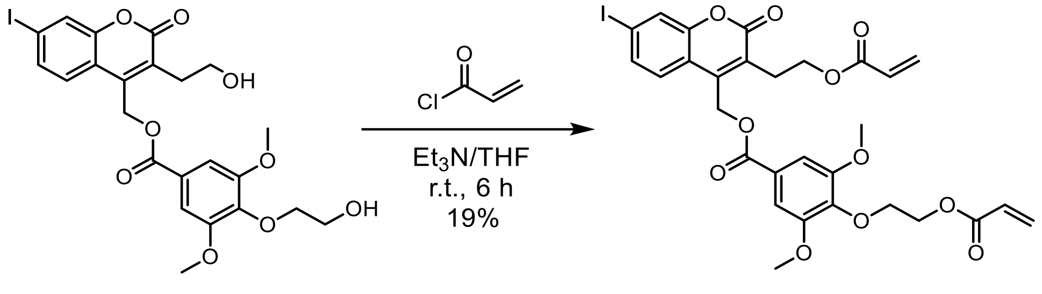

S5 **2b**

Under a nitrogen atmosphere, **S5** (295 mg, 0.517 mmol) and Et_3N (360 µl, 2.6 mmol) were dissolved into degassed THF (2.2 ml). After 10 min stirring at 0 °C, acryloyl chloride (121 µl, 1.5 mmol) was added dropwise to the solution. The reaction mixture was allowed to warm to room temperature and stirred for 6 h. The reaction mixture was diluted with hexane/EtOAc (1/1) and filtered through a Celite pad. The filtrate was then washed with brine. The aqueous layer was extracted with hexane/EtOAc (2/1). The combined organic layers were dried using $MgSO_4$, filtered, and concentrated. The residue was purified by column chromatography on silica gel (hexane:EtOAc = 1:1) to yield **2b** as a white solid (97.1 mg, 0.143 mmol, 19%).

ESI HR-MS: (m/z) 701.04902 ($[M+Na]^+$, $C_{29}H_{27}IO_{11}Na$, calcd. 701.04903).

^1H NMR (500 MHz, $CDCl_3$): δ_H = 7.74 (d, *J* = 1.6 Hz, 1H), 7.63 (dd, *J* = 8.5, 1.7 Hz, 1H), 7.48 (d, *J* = 8.5 Hz, 1H), 7.20 (s, 2H), 6.41-6.38 (m, 1H), 6.37-6.35 (m, 1H), 6.14 (dd, *J* = 17.3, 10.4 Hz, 1H), 6.04 (dd, *J* = 17.3, 10.5 Hz, 1H), 5.82 (dd, *J* = 10.4, 1.4 Hz, 1H), 5.78 (dd, *J* = 10.5, 1.3 Hz, 1H), 5.53 (s, 2H), 4.43 (dd, *J* = 7.9, 3.4 Hz, 5H), 4.31 (dd, *J* = 5.5, 3.8 Hz, 3H), 3.82 (s, 7H), 3.27 (t, *J* = 6.5 Hz, 2H).

^{13}C NMR (126 MHz, $CDCl_3$, r.t.): δ_C = 166.04, 165.85, 165.5, 160.4, 153.2, 152.6, 143.8, 141.5, 133.7, 131.3, 131.0, 128.3, 128.0, 126.8, 126.2, 126.0, 124.1, 118.2, 107.0, 96.6, 70.8, 63.7, 62.5, 59.2, 53.4, 27.4.

Synthesis of **3b**

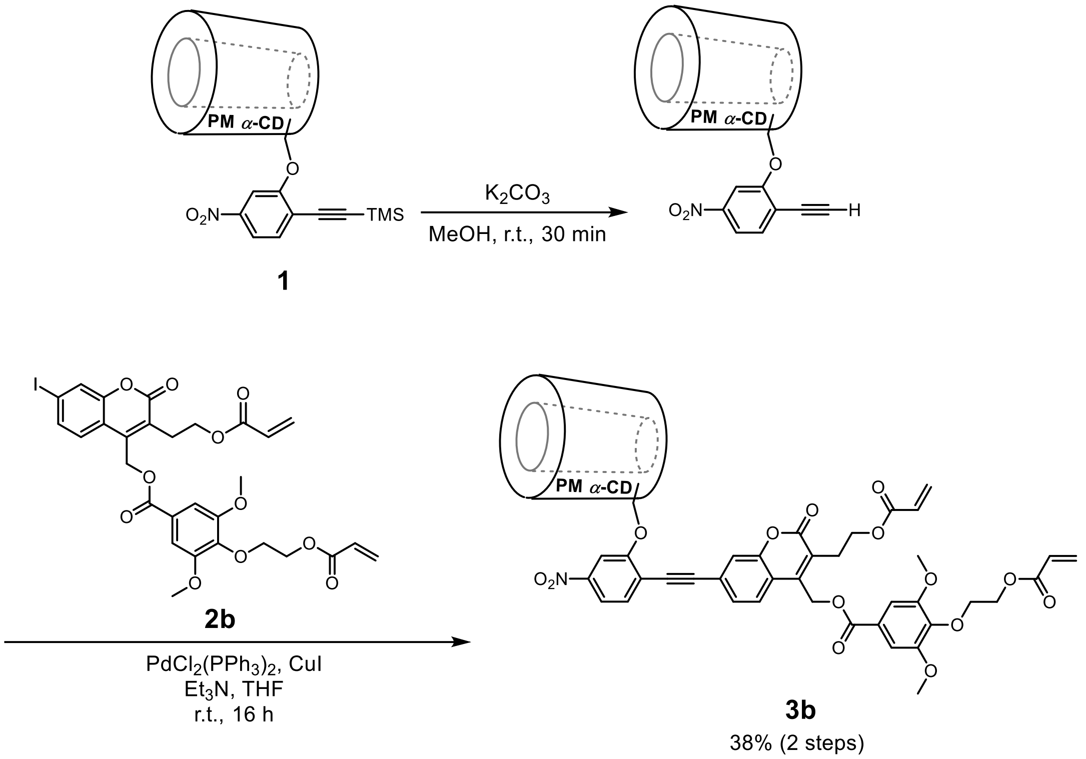

1 (293 mg, 0.205 mmol) and K_2CO_3 (85.5 mg, 0.619 mmol) were dissolved in MeOH (6.7 ml) under a nitrogen atmosphere. The reaction mixture was stirred at room temperature. After 30 min stirring, the mixture was dried *in vacuo* and then filtered through a Celite pad eluting with $CHCl_3$. The filtrate was concentrated, and the crude product was used immediately without further purification. The crude product was added into degassed THF (13.5 ml) and Et_3N (4.6 ml) and then stirred under nitrogen bubbling for 10 min. Under a nitrogen atmosphere, **2b** (139 mg, 0.205 mmol), CuI (1.5 mg, 7.9 μmol), and $PdCl_2(PPh_3)_2$ (7.5 mg, 11 μmol) were added into the solution, and then the reaction mixture was stirred at room temperature for 23 h. The reaction mixture was then washed with NH_4Cl (aq.) solution. The aqueous layer was extracted with CH_2Cl_2. The combined organic layers were dried using $MgSO_4$, filtered, and concentrated *in vacuo*. The residue was further purified by GPC with $CHCl_3$ as the eluent to yield **3b** as a yellow solid (150 mg, 78.7 μmol, 38%).

ESI HR-MS: (m/z) 1928.72051 ($[M+Na]^+$, $C_{90}H_{123}NO_{43}Na$, calcd. 1928.73610).

^1H NMR (500 MHz, $CDCl_3$): δ_H = 7.86 (dd, J = 8.4, 2.1 Hz, 1H), 7.81 (d, J = 2.0 Hz, 1H), 7.75 (d, J = 8.4 Hz, 1H), 7.65-7.63 (d and s, 2H), 7.51 (dd, J = 8.3, 1.6 Hz, 1H), 7.22 (s, 2H), 6.41 (dd, J = 9.1, 1.4 Hz, 1H), 6.37 (dd, J = 9.1, 1.4 Hz, 1H), 6.14 (dd, J = 17.3, 10.4 Hz, 1H), 6.06 (dd, J = 17.3, 10.5 Hz, 1H), 5.83 (dd, J = 10.4, 1.4 Hz, 1H), 5.79 (dd, J = 10.5, 1.3 Hz, 1H), 5.57 (s, 2H), 5.08-3.02 (m, 107H).

^{13}C NMR (126 MHz, $CDCl_3$, r.t.): δ_C = 166.24, 166.08, 165.7, 160.7, 159.9, 153.4 (several peaks overlapped), 152.8, 148.6, 143.8, 141.7, 133.8, 131.5, 131.2 (several peaks overlapped), 128.5 (several peaks overlapped), 128.2 (several peaks overlapped), 127.9, 127.4, 125.7, 125.3, 124.3, 120.3, 119.44, 119.28, 116.2, 107.23 (several peaks overlapped), 107.10, 100.66, 100.54, 100.46, 100.41, 100.38, 100.36, 96.8, 87.9, 82.94, 82.82, 82.77 (several peaks overlapped), 82.46 (several peaks overlapped), 82.42, 82.39 (several peaks overlapped), 82.27, 82.19, 81.41 (several peaks overlapped), 81.36, 81.25, 71.94, 71.89, 71.79, 71.74 (several peaks overlapped), 71.70, 71.56, 71.53, 71.50, 71.42, 71.05 (several peaks overlapped), 70.4, 68.7, 63.9 (several peaks overlapped), 62.9, 62.07, 62.05, 62.04, 62.01, 61.98, 59.55, 59.44, 59.33 (several peaks overlapped), 59.1, 58.5, 58.15, 58.10, 58.04, 58.03, 57.7, 56.5 (several peaks overlapped), 27.7.

Synthesis of **4b**

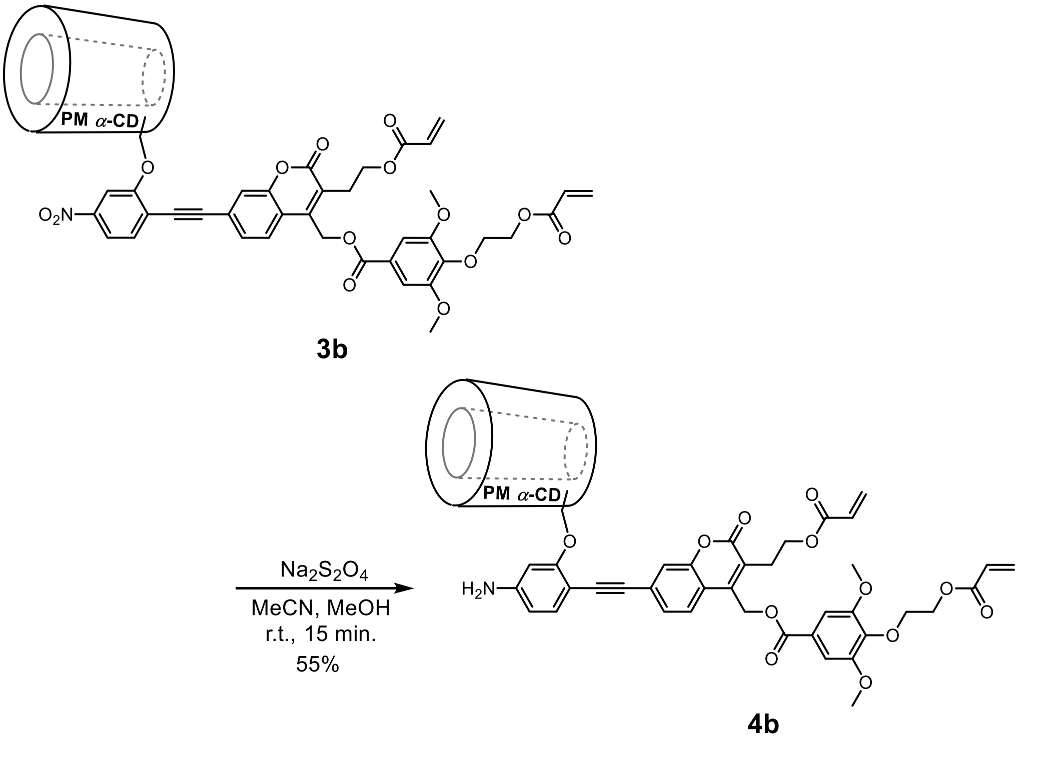

Under a nitrogen atmosphere, compound **3b** (150 mg, 78.7 μmol) and $Na_2S_2O_4$ (149 mg, 0.855 mmol) were dissolved into H_2O/MeCN (21.5/21.5 ml). After 15 min, the reaction mixture was diluted with brine. The aqueous layer was extracted with EtOAc. The combined organic layers were dried using $MgSO_4$, filtered, and concentrated. The residue was further purified by GPC with $CHCl_3$ as the eluent to yield **4b** as a yellow solid (80.9 mg, 43.1 μmol, 55%).

ESI HR-MS: (m/z) 1898.76030 ($[M+Na]^+$, $C_{90}H_{125}NO_{41}Na$, calcd. 1898.76192).

^{1}H NMR (500 MHz, $CDCl_3$): δ_H = 7.68 (d, *J* = 8.3 Hz, 1H), 7.56 (s, 1H), 7.44 (d, *J* = 8.3 Hz, 1H), 7.30 (s, 1H), 7.24 (s, 2H), 6.42 (d, *J* = 10.0 Hz, 1H), 6.39 (d, *J* = 10.0 Hz, 1H), 6.27 (d, *J* = 8.2 Hz, 1H), 6.22 (s, 1H), 6.16 (dd, *J* = 17.3, 10.4, 0.7 Hz, 1H), 6.07 (dd, *J* = 17.3, 10.5, 0.7 Hz, 1H), 5.84 (d, *J* = 10.4 Hz, 1H), 5.80 (d, *J* = 10.5 Hz, 1H), 5.57 (s, 2H), 5.15-3.08 (m, 109H).
^{13}C NMR (126 MHz, $CDCl_3$, r.t.): δ_C = 166.17, 166.03, 165.7, 161.10, 161.05, 153.3 (several peaks overlapped), 152.8, 149.2, 143.9, 141.5, 134.8, 131.4 (several peaks overlapped), 131.1 (several peaks overlapped), 128.4 (several peaks overlapped), 128.2 (several peaks overlapped), 127.9, 127.4, 125.9, 124.8, 124.4, 119.3, 117.8, 107.7, 107.1 (several peaks overlapped), 102.0, 100.48 (several peaks overlapped), 100.37, 100.27, 100.22, 100.0, 99.3, 91.6, 90.7, 83.0, 82.72, 82.67, 82.54, 82.48, 82.36, 82.32, 82.26, 82.16, 81.36, 81.31, 81.27, 81.24, 72.0, 71.64, 71.59 (several peaks overlapped), 71.55, 71.49 (several peaks overlapped), 71.44 (several peaks overlapped), 71.39, 71.0, 70.7, 70.3, 68.0, 63.8 (several peaks overlapped), 62.9, 62.04, 61.95 (several peaks overlapped), 61.89, 61.85, 59.46, 59.31, 59.22, 59.21, 59.14, 59.05, 58.3, 58.07, 58.04, 57.98, 57.95, 57.6, 56.4 (several peaks overlapped), 27.4.

S2.2. Synthesis of gels

General procedure

Gels were synthesized via thermal-initiated radical polymerization of the corresponding monomer and crosslinker. A pregel solution containing *N*-isopropylacrylamide (NIPAm), **4b**, *N*,*N'*-methylenebisacrylamide (MBA), and 2,2'-azobis(2,4-dimethylvaleronitrile) (ADVN) in DMSO, as shown in Table S1 was degassed three times via freeze-thaw technique and was filled into the gap between two PTFE coated glass slides with a 0.5 mm PTFE thick spacer. The glass slides and spacer were held with binder clips (Fig. S1). The reaction solution was then placed in an oven (60 °C, 18 h) for polymerization. After polymerization, the network material was washed with a large amount of acetone, followed by shrunk in hexane, and dried under a vacuum. The dried samples were reswollen with $CDCl_3$-EtOH (95/5, v/v) to yield gel samples (**G1** and **G2**).

Synthesis of **G1** and **G2**

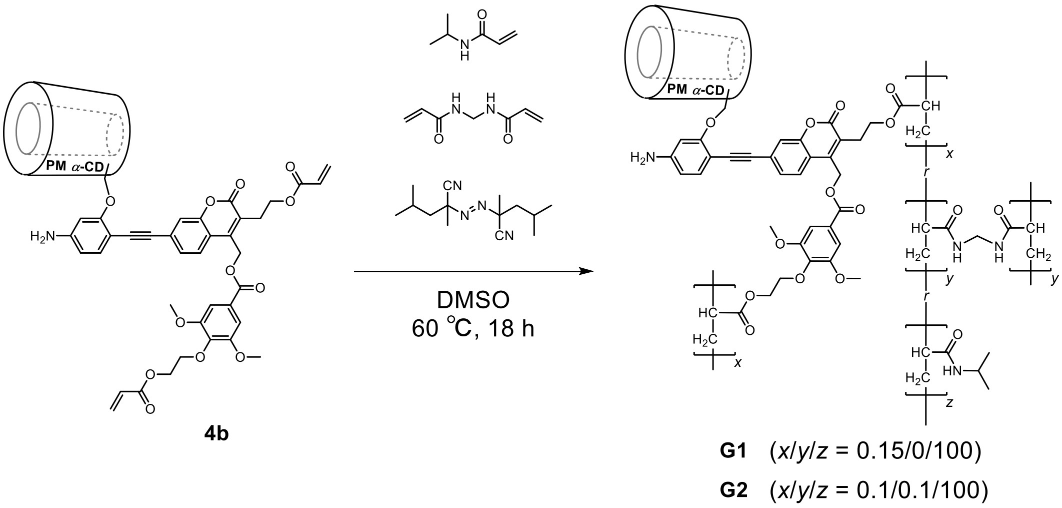

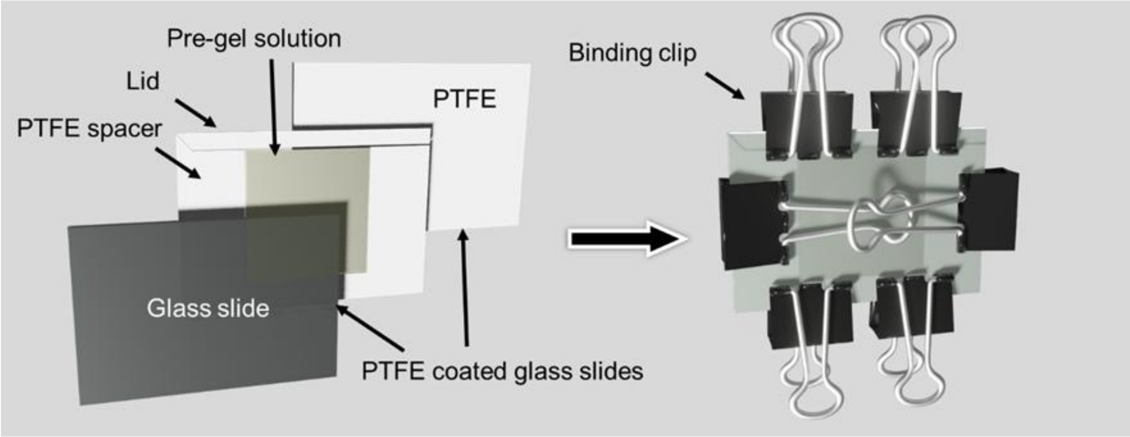

Fig. S1. Schematic representation of procedure adopted for preparing the gels.

Table S1. Summary of composition of the reaction solution for the synthesis of gels.

Gels	NIPAm		Crosslinker				ADVN		DMSO
			**4b**		MBA				
	[mg]	[mmol]	[mg]	[μmol]	[mg]	[μmol]	[mg]	[μmol]	[μL]
**G1**	151	1.3	3.8	2.0	—	—	3.0	13	173
**G2**	431	3.8	7.2	3.8	0.6	3.8	9.5	38	550

S3. Supramolecular conformational isomerization of 4a and 4a'

Compound **4a** underwent supramolecular conformational isomerization to its insulated counterpart (**4a'**) in CH_3OH-H_2O (1/1, v/v) as a hydrophilic solvent (Fig. 2a). The intramolecular insulation process originated from hydrophilic-hydrophobic interactions between the π-system of the ethynyl coumarinylmethyl ester derivatives and the inner protons of PM α-CD. After heating **4a** in CH_3OH-H_2O (1/1, v/v) at 40 °C, ^1H NMR analyses in $CDCl_3$ demonstrated that the generation of new signals attributable to **4a'** in **4a** gradually decreased, with **4a** being fully converted to **4a'** within 2 h (Fig. 2b). The aromatic proton ($H_{a–c}$) in **4a'** chemically shifted downfield from 7.5 to 8.0 ppm, as compared with that in **4a**, as a characteristic result of the quantitative insulation of the PM α-CD-based kinetically stable pseudo[1]rotaxane structure.[54,55] The proton signal of the coumarinylmethyl group (H_d) was split after insulation owing to the unique stereogenic nature of PM α-CD. The ROESY NMR spectrum of **4a'** exhibits a strong nuclear Overhauser effect between the π-system and the inner protons of PM α-CD, demonstrating the formation of a threading structure (Fig. S35). Furthermore, treating the insulated structure (**4a'**) in $CHCl_3$ at 60 °C for 10 h quantitatively converted it back to its uninsulated counterpart (**4a**), demonstrating the reversible transformation between the insulated and uninsulated structures (Fig. S2).

S3.1. Experimental procedures

*Uninsulation of **4a'***

A solution of **4a'** (2.0 mg, 1.1 μmol) in $CDCl_3$ (750 μL) was heated at 60 °C. The ^1H NMR spectra were measured after 0–600 min, indicating the gradual uninsulation of **4a'** to form **4a** (Fig. S2).

*Insulation of **4a***

A solution of **4a** (2.0 mg, 1.1 μmol) in CH_3OH-H_2O (1/1, v/v, 400 μL) was heated at 40 °C. After 0, 20, and 120 min, the solvent was dried in a vacuum. The residue was dissolved in $CDCl_3$. The ^1H NMR spectra were measured to indicate the gradual insulation of **4a** to form **4a'** (Fig. 2b).

Kinetic stability of

*Kinetic stability of **4a** and **4a'***

The threading/dethreading processes between **4a** and **4a'** occur on the terminal side with

the amino group through the flipping motion of the PM α-CDs, accompanied by rotation of the 1,6-glycosidic bonds of the cyclodextrins.[56,57] The relative position between the cyclic and axle molecules in the kinetically stable pseudo[1]rotaxane structure is constrained by the covalent linkage. Consequently, the amino group at the axle terminal significantly influences the threading/dethreading rate owing to steric hindrance, which increases the activation barrier,[58,59] enabling kinetic stabilization of both the insulated and uninsulated structures. In particular, the ^{1}H NMR spectra (Fig. S3 and S4) confirmed that **4a** and **4a'** remained stable at r.t. after 6 h, even in thermodynamically unfavorable solvents, including $CDCl_3$ (a low polarity solvent for **4a'**) and CD_3OD (a high polarity solvent for **4a**). Therefore, the supramolecular conformational isomers (**4a** and **4a'**) demonstrated kinetic stability under various solvent polarities, allowing systematic investigations of the insulation effects on photoreactivity by comparing the insulated and uninsulated molecules in the same solvents with various polarities. Accordingly, the kinetically stable supramolecular conformational isomers (**4a** and **4a'**) were quantitatively and reversibly switchable by heating the solution with low/high-polarity solvents, which is attributed to the kinetically stable pseudo[1]rotaxane structure.

To confirm the kinetic stability at room temperature, **4a** and **4a'** (3.0 mg, 1.6 μmol) were dissolved in CD_3OD (as a hydrophilic solvent) and $CDCl_3$ (as a hydrophobic solvent), respectively. The supramolecular structures of **4a** and **4a'** remained stable even after 6 h at room temperature, which was confirmed by ^{1}H NMR spectra (Fig. S3 and S4).

*Repetitive conformational isomerization of **4a** and **4a'***

Repetitive transformation cycles were monitored by measuring emission spectra in $CHCl_3$ (Fig. 2d). Each cycle involved the following two steps: (1) A solution of **4a** in CH_3OH-H_2O (1/1, v/v) was heated for 4.5–7.5 h to form **4a'**. (2) A solution of **4a'** in $CHCl_3$ was heated overnight to form **4a**.

S3.2. Kinetic trace of 4a'

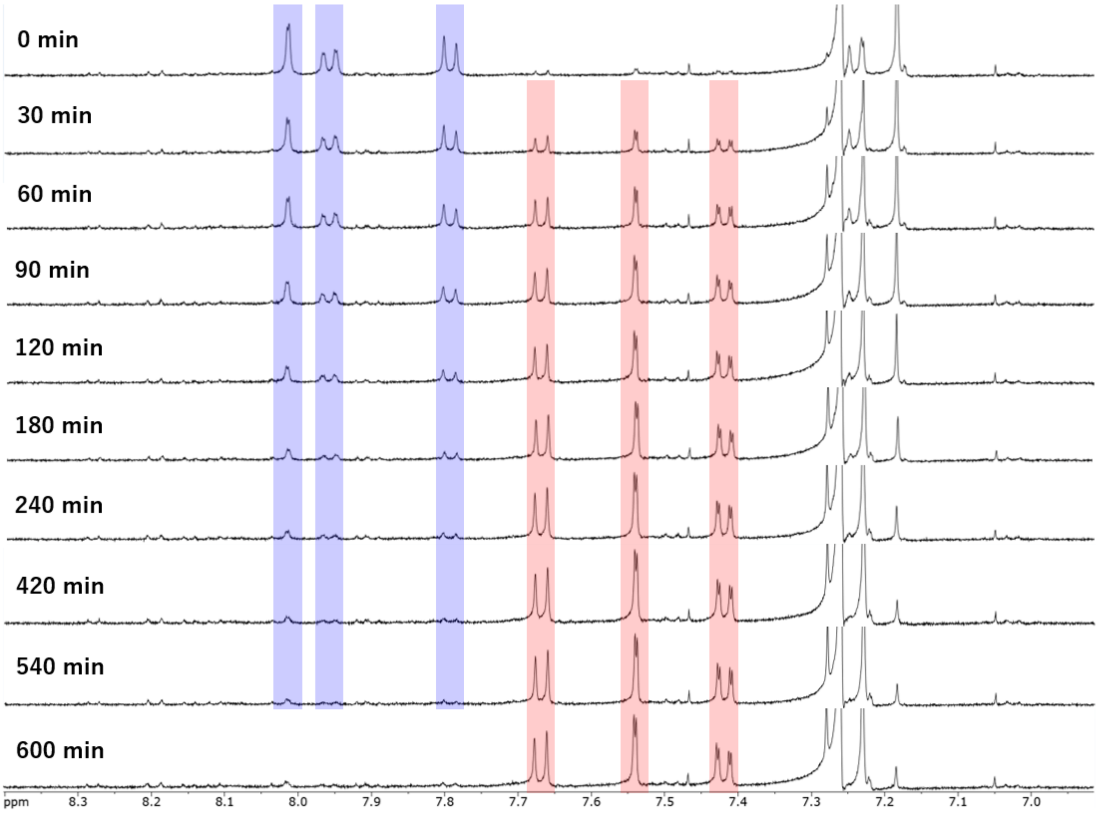

Fig. S2. Time evolution of the ^{1}H NMR spectra ($CDCl_3$, 500 MHz, r.t.) showing the conversion of **4a'** to **4a** in $CDCl_3$ at 60 °C. The red area shows **4a** and the blue area shows **4a'**.

S3.3. Kinetic stability of 4a and 4a'

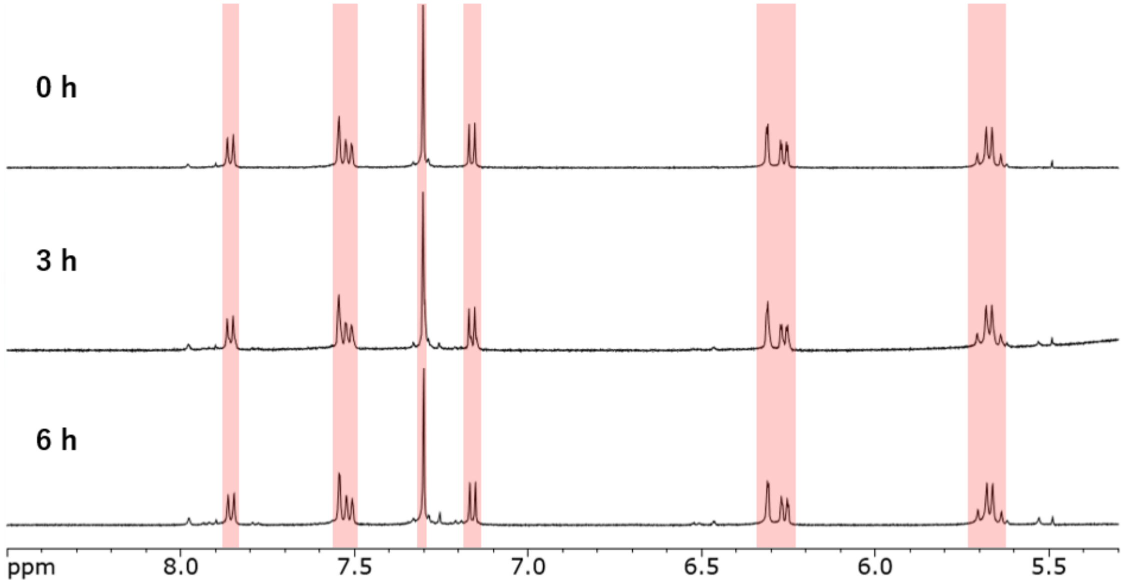

Fig. S3. ^{1}H NMR spectra ($CDCl_3$, 500 MHz, r.t.) of **4a** after 0 h, 3 h, and 6 h in CD_3OD at room temperature.

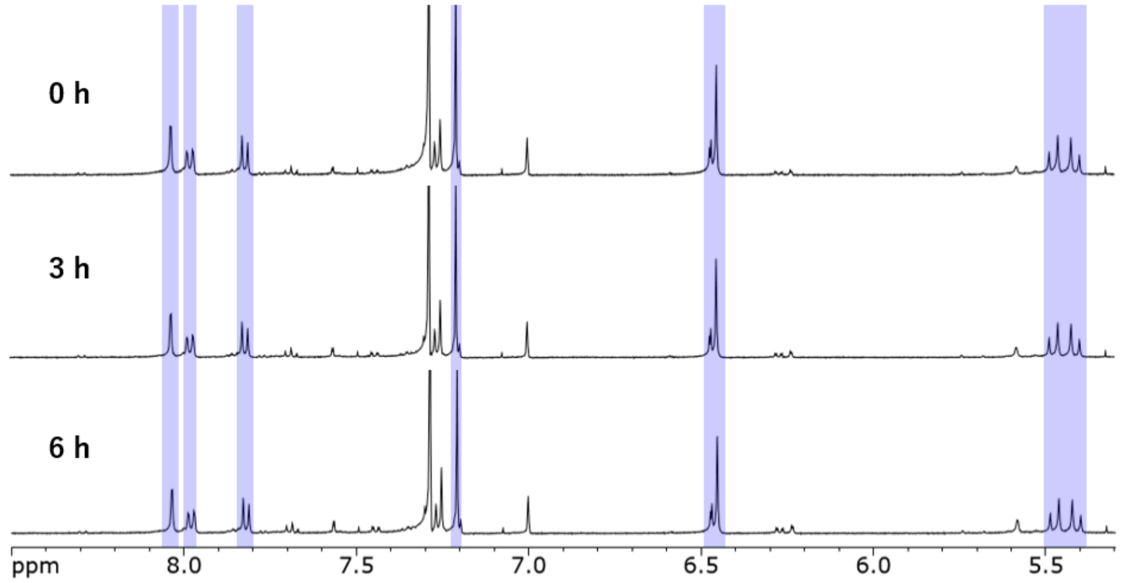

Fig. S4. ^{1}H NMR spectra ($CDCl_3$, 500 MHz, r.t.) of **4a'** after 0 h, 3 h, and 6 h in $CDCl_3$ at room temperature.

S4. Photodegradation measurements of 4a and 4a'

S4.1. Evaluation of photodegradation rate of 4a and 4a'

The photodegradation of **4a** and **4a'** was investigated by ^{1}H NMR analyses. A solution of **4a** and **4a'** (5.0 mg, 2.7 µmol) in $CDCl_3$-EtOH (95:5, v/v) was prepared, with 1,3,5-trimethoxybenzene (454 µg, 2.7 µmol) added as an internal standard for ^{1}H NMR analysis. After N_2 bubbling, the solution was irradiated with UV light (365 nm, 480 mW cm^{-2}) at room temperature. After the irradiation, ^{1}H NMR spectra were recorded to assess degradation by comparing the integrated values of the protons H*e* of **4a** and **4a'** (Fig. 3a) before and after exposure (Fig. S5 and S6).

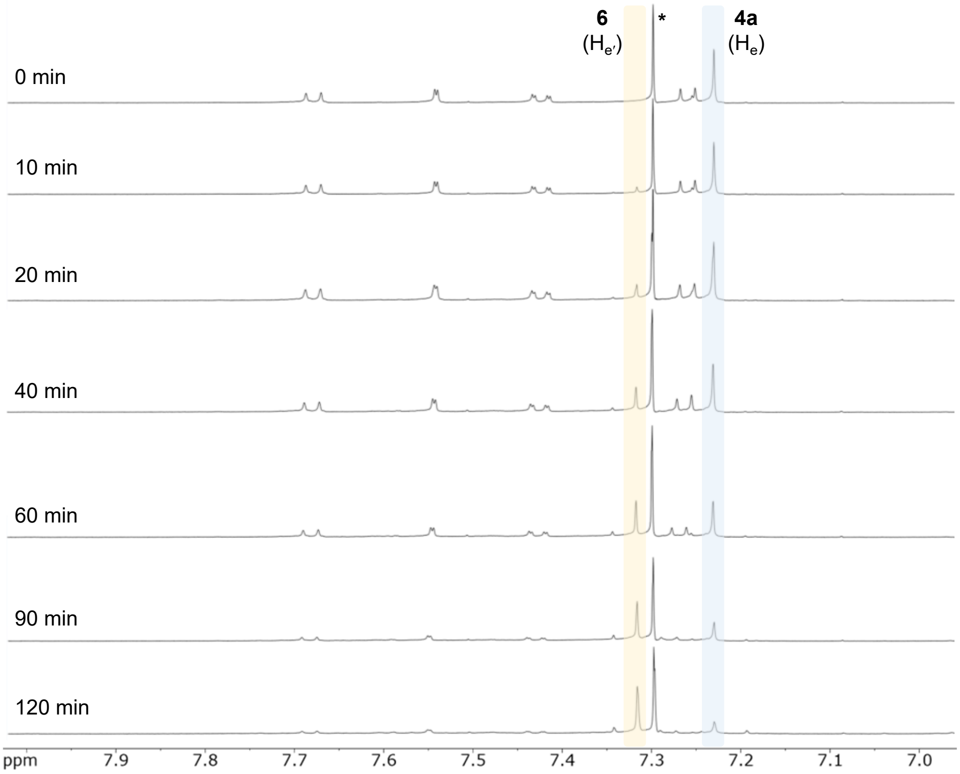

Fig. S5. Aromatic region of ^{1}H NMR spectra ($CDCl_3$-EtOH (95/5, v/v), 500 MHz, r.t.) recorded before and after the photoirradiation of **4a** (5.0 mg, 2.7 µmol) in $CDCl_3$-EtOH (95/5, v/v). Asterisk: $CHCl_3$.

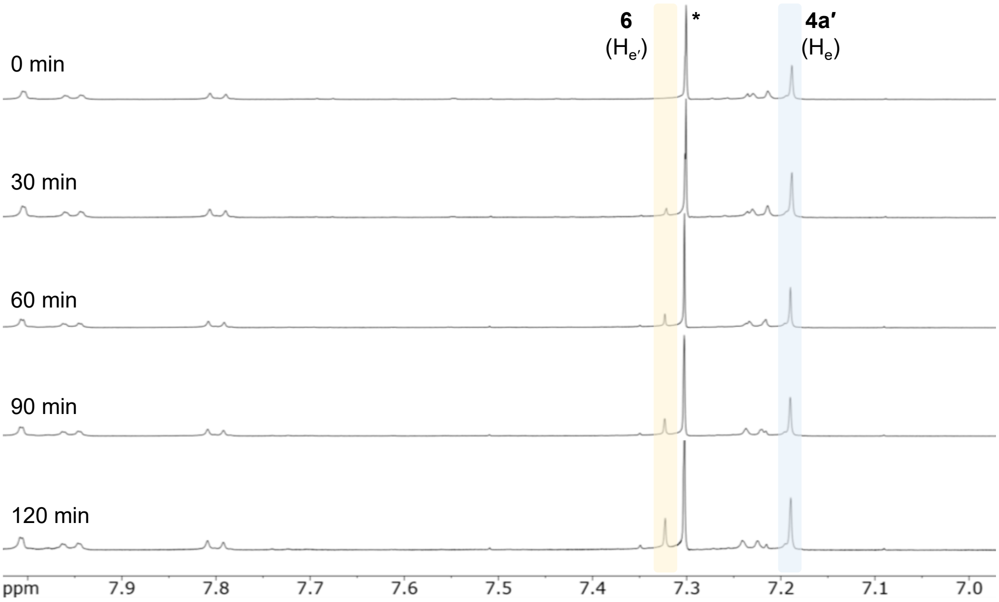

Fig. S6. Aromatic region of ^{1}H NMR spectra ($CDCl_3$-EtOH (95/5, v/v), 500 MHz, r.t.) recorded before and after the photoirradiation of **4a'** (5.0 mg, 2.7 µmol) in $CDCl_3$-EtOH (95/5, v/v). Asterisk: $CHCl_3$.

S4.2. Solvent dependency of photodegradation rate

The photodegradation of **4a** and **4a'** was investigated by ^{1}H NMR analyses. A solution of **4a** and **4a'** (0.5 mg, 0.27 µmol) in $CDCl_3$-EtOH (95:5, v/v) or DMSO-d_6 (750 µL) was prepared, with 1,3,5-trimethoxybenzene (91 µg, 0.54 µmol) added as an internal standard for ^{1}H NMR analysis. The solution was irradiated with UV light (365 nm, 480 mW cm^{-2}) at room temperature for 10 min. After the irradiation, ^{1}H NMR spectra were recorded to assess degradation by comparing the integrated values of the protons H*e* of **4a** and **4a'** (Figure 3a) before and after exposure (Figures S7, S8).

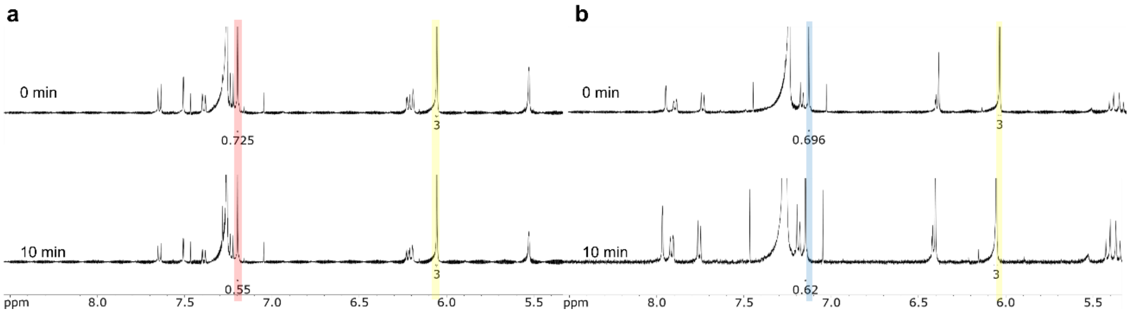

Fig. S7. Aromatic region of ^{1}H NMR spectra ($CDCl_3$-EtOH (95/5, v/v), 500 MHz, r.t.) recorded before and after the photoirradiation of (a) **4a** (red) and (b) **4a'** (blue) in $CDCl_3$-EtOH (95/5, v/v). The red, blue, and yellow regions correspond to **4a**, **4a'**, and 1,3,5-

trimethoxybenzene as an internal standard.

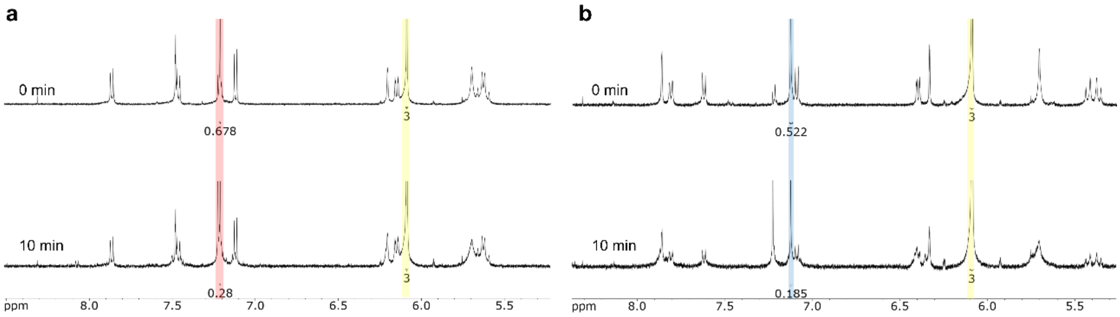

Fig. S8. Aromatic region of ^{1}H NMR spectra (DMSO-d_6, 500 MHz, r.t.) recorded before and after the photoirradiation of (a) **4a** (red) and (b) **4a'** (blue) in DMSO-d_6. The red, blue, and yellow regions correspond to **4a**, **4a'**, and 1,3,5-trimethoxybenzene as an internal standard.

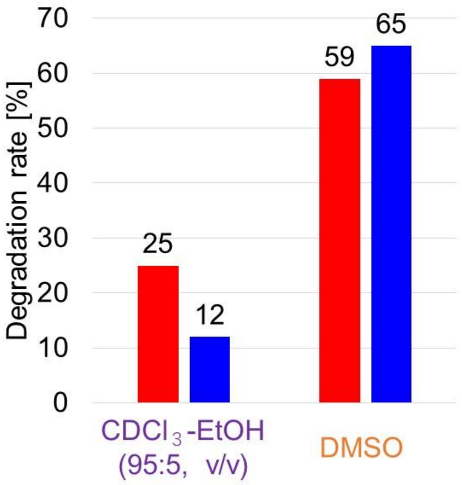

Fig. S9. Photodegradation ratio of **4a** (red) and **4a'** (blue) under $CHCl_3$-EtOH (95/5, v/v) and DMSO.

S5. Identification of photodegraded product

4a', which was prepared from **4a** (5.1 mg, 2.8 µmol) via insulation process with CH_3OH-H_2O, was dissolved in EtOH (16 mL). The solution was exposed to UV light (365 nm, 480 mW cm^{-2}) for 45 min at room temperature. After the reaction, ESI-MS were measured to confirm **5a** (or **5a'**) and **6** as photodegraded products, as shown in Fig. S10.

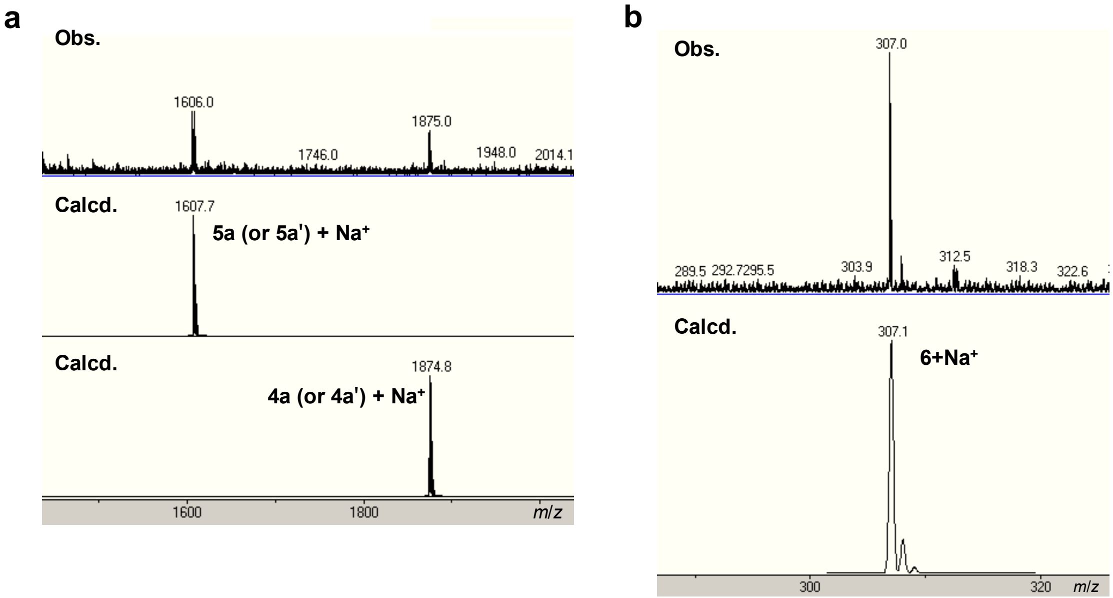

Fig. S10. Mass spectra of crude product after photoirradiation (365 nm, 480 mW cm^{-2}) of **4a'** in $CDCl_3$-EtOH (95/5, v/v) with the calculated mass of **4a**, **5a**, and **6**.

S6. Measurements of swelling ratio of G1 and G1'

The swelling ratio of **G1** and **G1'** was assessed in $CHCl_3$, $CHCl_3$-EtOH (95/5, v/v), and MeOH-H_2O (5/2, v/v). These gels were immersed in the solvent (2 ml) for 3 h to equilibrate the swelling. The weight of the swollen gels was measured after the excess surface solution was blotted with weighing paper. These gels were then washed with acetone and dried under vacuum for 18 h. The mass of the resulting dried gels was measured. Each experiment was conducted three times; the date and error bars represent averages and standard error, respectively.

S7. Supramolecular conformational isomerization of G1 and G1'

*Uninsulation of **G1'***

Dried **G1'** was immersed in $CHCl_3$ at 60 °C for 4 h. The gel was shrunk in hexane, and dried under a vacuum. The dried samples were reswollen with $CHCl_3$-EtOH (95/5, v/v) to yield gel samples (**G1**).

*Insulation of **G1***

Dried **G1** was immersed in CH_3OH-H_2O (5/2, v/v) at 60 °C for 3–15 h. The gel was washed with a large amount of acetone, followed by shrunk in hexane, and dried under a vacuum. The dried samples were reswollen with $CHCl_3$-EtOH (95/5, v/v) to yield gel samples (**G1'**).

S8. Kinetic trace of G1 and G1'

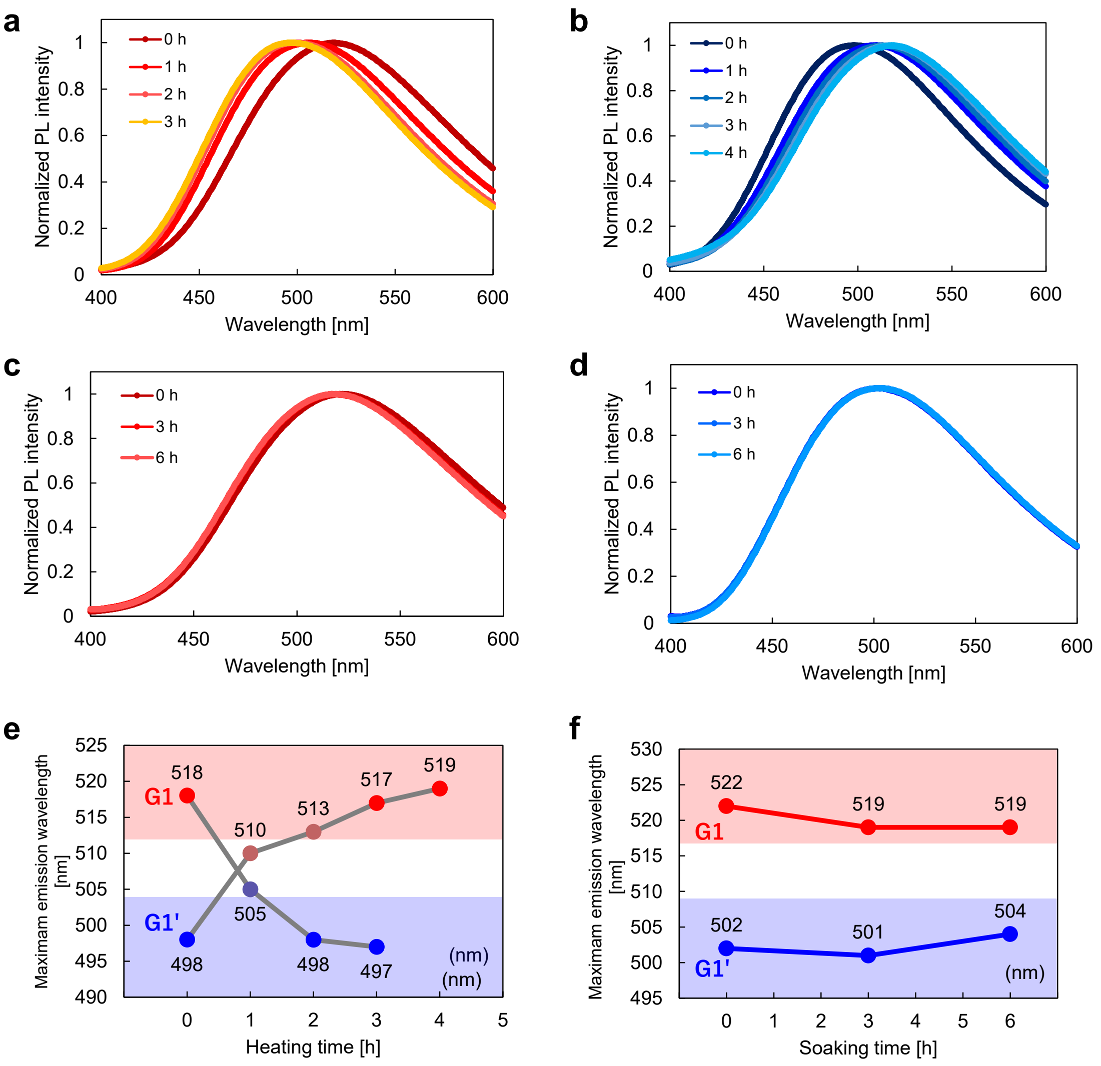

Fig. S11. (a,b) Emission spectra of (a) **G1** in CH_3OH-H_2O (5/2, v/v) and (b) **G1'** in $CHCl_3$ at 60 °C. (c,d) Emission spectra of (a) **G1** in CH_3OH-H_2O (5/2, v/v) and (b) **G1'** in $CHCl_3$ at room temperature. (e,f) Time-evolution of maximum emission wavelength of **G1** in CH_3OH-H_2O (5/2, v/v) and **G1'** in $CHCl_3$ at (e) 60 °C and (f) room temperature (excitation at 365 nm).

S9. Photodegradability of G1 and G1'

The photodegradation reaction of **G1** and **G1'** was evaluated by tracing the dynamic viscoelasticity during 365 nm UV light irradiation. For the photodegradation reaction, dried gels were immersed in 2 ml of $CHCl_3$-EtOH (95/5, v/v) or DMSO for 3 h to equilibrate the swelling and were exposed to UV light at room temperature. The irradiation initiated 1 min after the start of rheology measurement (Fig. 4e and S12).
The periodic ON/OFF cycles of the UV light irradiation (365 nm, 33 mW cm^{-2}) demonstrated that the decrease in G' was directly induced by the photoirradiation (Fig. S13). Moreover, the decrease in G' accelerated as the UV irradiation intensity increased (365 nm, 8–132 mW cm^{-2}) (Fig. S14), indicating that the softening of **G1** was derived from photoexcitation with 365 nm UV light.

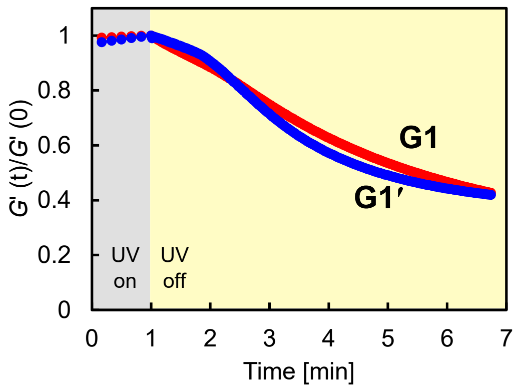

Fig. S12. Time-evolution of normalized storage modulus during photoirradiation (365 nm, 33 mW cm^{-2}) of **G1** and **G1'** under DMSO.

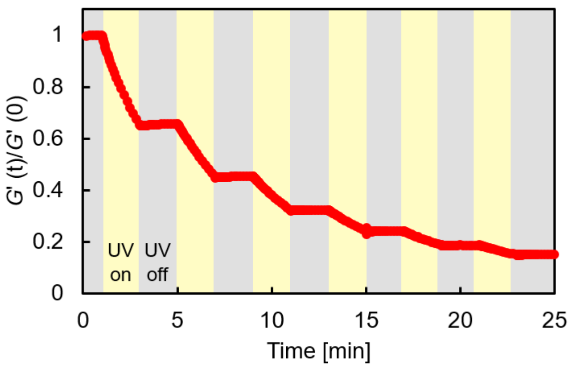

Fig. S13. Time-evolution of normalized storage modulus of **G1** under $CHCl_3$-EtOH (95/5, v/v) during photoirradiation ON/OFF switching.

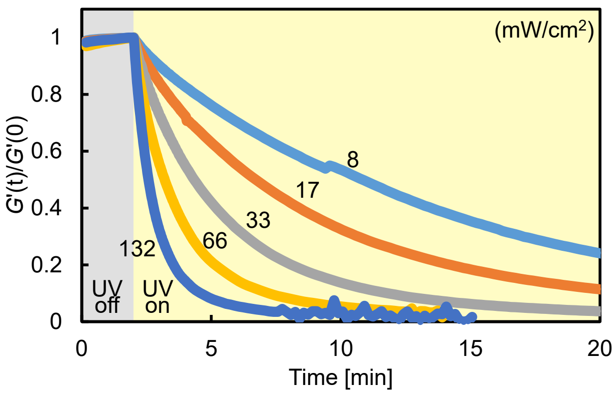

Fig. S14. Time-evolution of normalized storage modulus of **G1** during photoirradiation at various light intensities under $CHCl_3$-EtOH (95/5, v/v).

S10. Macroscopic photodegradation

G1 and **G1'** immersed in 3 ml of $CHCl_3$-EtOH (95/5, v/v) in lidded Petri dish. The gels were exposed to UV light (CCS, HLDL-50U6-NWPSC, 365 nm, 5 cm square, 116 mW cm^{-2}) for 20 min (Fig. S15). The macroscopic photodegradability of **G1** and **G1'** successfully reflected the nanoscale molecular reactivity of **4a** and **4a'**.

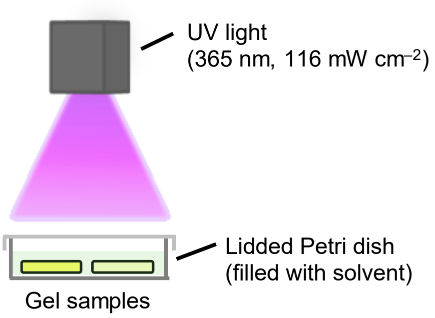

Fig. S15. A procedure of light irradiation for macroscopic degradation of **G1** and **G1'**.

S11. Computational calculations

The structural optimization of **S6** as a model structure of insulated coumarinylmethyl derivatives was determined using DFT calculation under B3LYP/6-31G(d,p) level, performed with Gaussian 16 software (Fig. S16).[4] The optimized geometry is presented in the following Table S2.

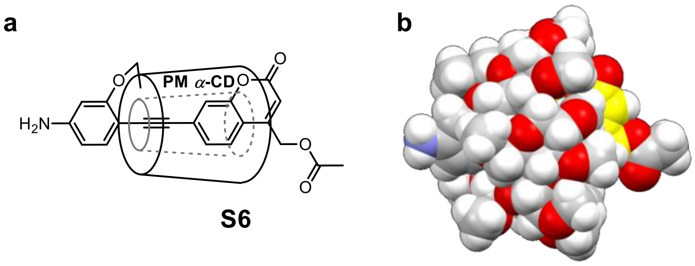

Fig. S16. (a) Chemical structure and (b) calculated structure with a space-filling model of **S6** (carbon atoms on coumarinylmethyl moiety were colored in yellow for clarity).

Table S2. Optimum structural coordinates of **S6** by B3LYP/ 6-31G(d,p). stoichiometry $C_{73}H_{107}NO_{34}$.

Num.	Atom	Coordinates / Å		
		X	Y	Z
1	C	-1.0139	1.0164	-0.4403
2	C	-0.9601	2.1252	-1.2659
3	C	0.2457	2.5338	-1.8763
4	C	1.3937	1.7622	-1.6099
5	C	1.3626	0.6476	-0.7801
6	C	0.1537	0.2520	-0.1831
7	C	0.3895	3.6758	-2.7575
8	C	1.6042	3.9610	-3.2931
9	C	2.7846	3.1618	-3.0116
10	O	2.6057	2.0714	-2.1670
11	O	3.9013	3.3615	-3.4402
12	C	0.1067	-0.9000	0.6500
13	C	-0.8287	4.5117	-3.0437
14	O	-0.4873	5.5885	-3.9219
15	C	0.0924	-1.9011	1.3490
16	C	0.0352	-3.0809	2.1369
17	C	-0.9662	-3.2451	3.1169
18	C	-1.0540	-4.4001	3.8773
19	C	-0.1276	-5.4461	3.7008
20	C	0.8902	-5.2855	2.7465
21	N	-0.1829	-6.5848	4.4912
22	C	0.9751	-4.1292	1.9810
23	C	-1.5144	6.4232	-4.2403
24	O	-2.6379	6.2731	-3.8174
25	H	-1.9564	0.7289	0.0111
26	H	-1.8722	2.6848	-1.4453
27	H	2.2783	0.0935	-0.6195
28	H	1.7509	4.8049	-3.9527
29	H	-1.6178	3.9029	-3.5008
30	H	-1.2467	4.9158	-2.1141
31	H	-1.6528	-2.4282	3.3056
32	H	-1.8357	-4.4966	4.6259
33	H	1.6333	-6.0615	2.5903
34	H	0.2807	-7.4040	4.1255
35	H	-1.0801	-6.7938	4.9052
36	C	-4.1684	-1.6957	0.5711
37	C	-5.0393	-0.4259	0.4034
38	C	-5.5716	-0.1435	-1.0147
39	O	-4.5473	-2.7740	-0.3089
40	C	-4.2998	-2.2613	1.9886
41	O	-5.6490	-2.5294	2.3494
42	O	-4.2857	0.7242	0.8511
43	O	-6.6939	0.7024	-0.7948
44	O	-6.5850	-1.3451	-2.9680
45	C	-7.2098	1.4122	-1.9097
46	C	-5.8611	-0.8086	-4.0701
47	C	-4.7413	-2.4341	-1.6671
48	C	-5.9428	-1.4657	-1.7107
49	C	-1.7003	-3.5483	-2.2060
50	C	-2.6886	-2.6853	-3.0277
51	C	-1.9362	-1.6860	-3.9290
52	O	-0.7527	-4.1482	-3.1090
53	C	-2.3493	-4.7175	-1.4649
54	O	-1.4077	-5.4410	-0.6834
55	O	-3.5772	-1.8669	-2.2394
56	O	-2.8669	-1.2934	-4.9353
57	O	0.1174	-1.3129	-5.2852
58	C	-2.6986	-0.0063	-5.5034
59	C	0.5523	-0.1690	-4.5548
60	C	0.1430	-3.2457	-3.7305
61	C	-0.6802	-2.2751	-4.6044
62	C	2.5523	-3.1203	-1.0486
63	C	2.2630	-3.1594	-2.5575
64	C	3.3119	-2.4126	-3.4018
65	O	3.9326	-3.4122	-0.7875
66	C	1.7560	-4.1623	-0.2861
67	O	2.0303	-4.0105	1.1151
68	O	0.9628	-2.5860	-2.7876
69	O	3.1210	-2.9101	-4.7215
70	O	5.7585	-2.0693	-3.6660
71	C	3.5685	-2.0934	-5.7943
72	C	5.8745	-0.6453	-3.6516
73	C	4.8452	-2.4884	-1.3599
74	C	4.7430	-2.6769	-2.8919

75	C	4.4947	-0.9308	1.5416
76	C	5.2672	-0.6382	0.2224
77	C	5.5087	0.8575	-0.0743
78	O	4.8730	-0.0344	2.5986
79	C	4.8433	-2.3197	2.0643
80	O	4.0812	-2.6164	3.2194
81	O	4.5899	-1.1614	-0.9478
82	O	6.7110	0.8740	-0.8478
83	O	5.7824	3.1086	0.9632
84	C	6.9505	2.0254	-1.6548
85	C	4.7668	3.7422	0.1897
86	C	5.6810	1.7122	1.1952
87	C	1.4754	1.3079	3.6372
88	C	2.6212	2.2527	3.2175
89	C	2.1544	3.6710	2.8318
90	O	0.5832	1.9847	4.5441
91	C	2.0094	0.1193	4.4191
92	O	0.9504	-0.7782	4.6985
93	O	3.3688	1.7111	2.1088
94	O	3.3253	4.4644	3.0198
95	O	0.4182	5.4170	3.2831
96	C	3.3157	5.8017	2.5443
97	C	0.0180	5.5525	1.9245
98	C	-0.0833	3.1128	4.0255
99	C	0.9844	4.1804	3.6981
100	C	-2.7061	1.1104	3.2804
101	C	-2.3310	2.6098	3.1902
102	C	-3.1024	3.3910	2.1054
103	O	-4.1281	0.9258	3.1912
104	C	-2.3199	0.5324	4.6355
105	O	-2.6713	-0.8421	4.6776
106	O	-0.9257	2.7980	2.9309
107	O	-3.0421	4.7439	2.5555
108	O	-5.2865	3.5309	0.8895
109	C	-3.3994	5.7642	1.6369
110	C	-4.7477	3.4766	-0.4248
111	C	-4.7483	1.3907	2.0147
112	C	-4.5631	2.9209	1.9526
113	C	4.7186	1.3462	2.3445
114	H	-3.1205	-1.4308	0.3825
115	H	-5.9252	-0.5491	1.0345
116	H	-4.7990	0.3816	-1.5942
117	H	-3.6858	-3.1681	2.0653
118	H	-3.9293	-1.5270	2.7072
119	H	-6.4119	1.9171	-2.4734
120	H	-7.7656	0.7601	-2.5898
121	H	-7.8806	2.1743	-1.5043
122	H	-5.4186	0.1699	-3.8461
123	H	-5.0628	-1.4680	-4.4133
124	H	-6.5980	-0.6789	-4.8674
125	H	-5.0035	-3.3696	-2.1759
126	H	-6.7059	-1.9480	-1.0905
127	H	-1.1757	-2.9101	-1.4844
128	H	-3.2804	-3.3477	-3.6760
129	H	-1.6725	-0.8274	-3.2958
130	H	-3.1039	-4.3324	-0.7774
131	H	-2.8387	-5.3785	-2.1963
132	H	-1.7994	0.0533	-6.1257
133	H	-3.5773	0.1740	-6.1282
134	H	-2.6576	0.7739	-4.7284
135	H	0.8602	-0.4176	-3.5388
136	H	-0.2213	0.6086	-4.5165
137	H	1.4076	0.2348	-5.1024
138	H	0.7670	-3.8534	-4.3891
139	H	-1.0679	-2.9101	-5.4088
140	H	2.3007	-2.1314	-0.6590
141	H	2.2702	-4.2054	-2.8919
142	H	3.0876	-1.3386	-3.3595
143	H	2.0783	-5.1657	-0.5966
144	H	0.6892	-4.0626	-0.4903
145	H	4.6582	-2.1137	-5.8907
146	H	3.1102	-2.4952	-6.7011
147	H	3.2390	-1.0531	-5.6726
148	H	6.5682	-0.4010	-4.4603
149	H	4.9222	-0.1385	-3.8493
150	H	6.2718	-0.2862	-2.7023
151	H	5.8385	-2.8036	-1.0195
152	H	4.9249	-3.7485	-3.0327
153	H	3.4158	-0.8572	1.3626
154	H	6.2620	-1.0992	0.2892
155	H	4.6659	1.2224	-0.6744
156	H	4.6469	-3.0632	1.2848
157	H	5.9200	-2.3287	2.3091
158	H	7.1769	2.9044	-1.0437
159	H	7.8202	1.7840	-2.2713
160	H	6.0992	2.2442	-2.3122
161	H	3.7657	3.4323	0.4927
162	H	4.8835	4.8120	0.3726
163	H	4.8931	3.5634	-0.8830
164	H	6.6648	1.4430	1.5964
165	H	0.9235	0.9659	2.7554
166	H	3.2901	2.3791	4.0785
167	H	1.8611	3.6532	1.7721
168	H	2.8089	-0.3797	3.8598
169	H	2.4442	0.5005	5.3601
170	H	2.5981	6.4284	3.0830
171	H	4.3270	6.1841	2.7027
172	H	3.0941	5.8502	1.4692
173	H	-0.5259	4.6779	1.5654
174	H	-0.6410	6.4219	1.8944
175	H	0.8730	5.7479	1.2645
176	H	-0.6957	3.4938	4.8504
177	H	1.4257	4.4085	4.6753
178	H	-2.2017	0.5592	2.4780
179	H	-2.5899	3.0892	4.1438
180	H	-2.5587	3.2752	1.1566
181	H	-1.2472	0.6596	4.8162
182	H	-2.8826	1.0815	5.4096
183	H	-4.4556	5.7183	1.3557
184	H	-3.1998	6.7117	2.1438
185	H	-2.7841	5.7250	0.7256
186	H	-5.5636	3.7571	-1.0939
187	H	-3.9342	4.2008	-0.5668
188	H	-4.3961	2.4773	-0.6864
189	H	-5.8139	1.1816	2.1446
190	H	-5.0778	3.2788	2.8515
191	H	5.0730	1.8474	3.2507
192	C	-0.9107	-6.6229	-1.2903
193	H	-1.7249	-7.3282	-1.5174
194	H	-0.3584	-6.4134	-2.2126
195	H	-0.2415	-7.0899	-0.5627
196	C	4.3784	-3.9055	3.7156
197	H	3.7499	-4.0723	4.5934
198	H	4.1605	-4.6837	2.9701
199	H	5.4353	-3.9908	4.0154
200	C	1.3983	-1.8754	5.4737
201	H	1.7553	-1.5491	6.4643
202	H	0.5509	-2.5509	5.6017
203	H	2.2108	-2.4097	4.9671
204	C	-6.0914	-3.8556	2.1144
205	H	-6.1199	-4.1058	1.0484
206	H	-5.4488	-4.5882	2.6266
207	H	-7.1000	-3.9276	2.5308
208	C	-2.5276	-1.3828	5.9759
209	H	-1.4942	-1.2980	6.3391
210	H	-3.1948	-0.8813	6.6938
211	H	-2.8046	-2.4383	5.9216
212	C	-1.0479	7.5184	-5.1644
213	H	-0.6346	7.0862	-6.0802
214	H	-1.8874	8.1690	-5.4054
215	H	-0.2514	8.0972	-4.6880

S12. NMR spectra

S12.1. ^{1}H NMR and $^{13}C\{^{1}H\}$ NMR spectra

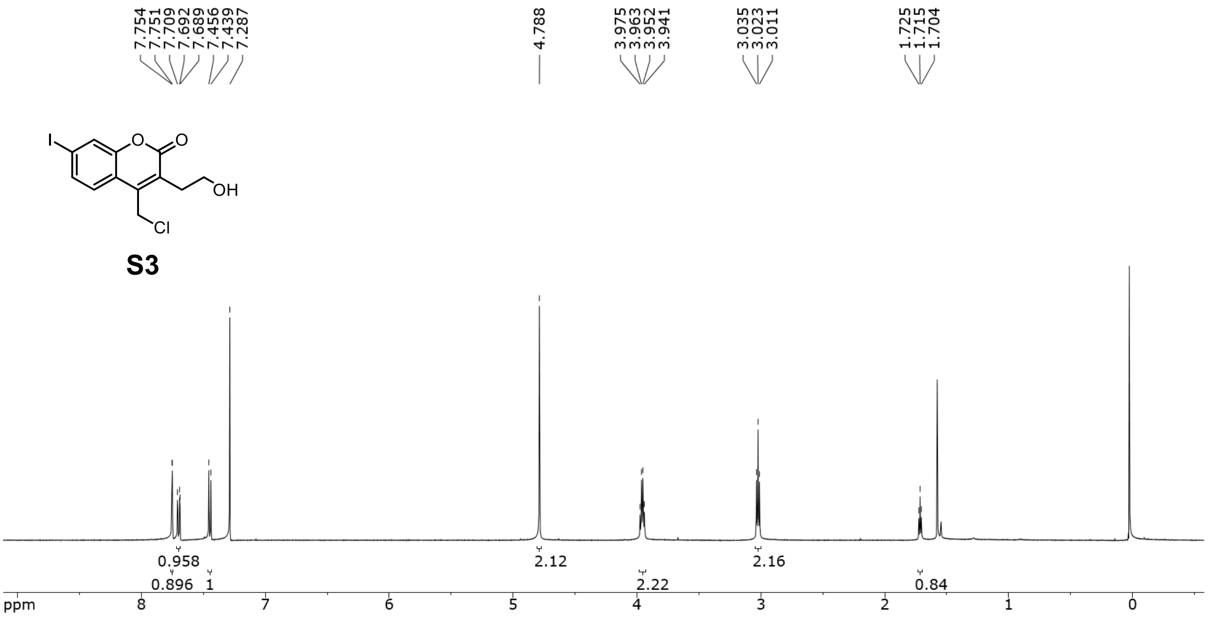

Fig. S17. ^{1}H NMR spectrum of **S3** (500 MHz, $CDCl_3$, r.t.).

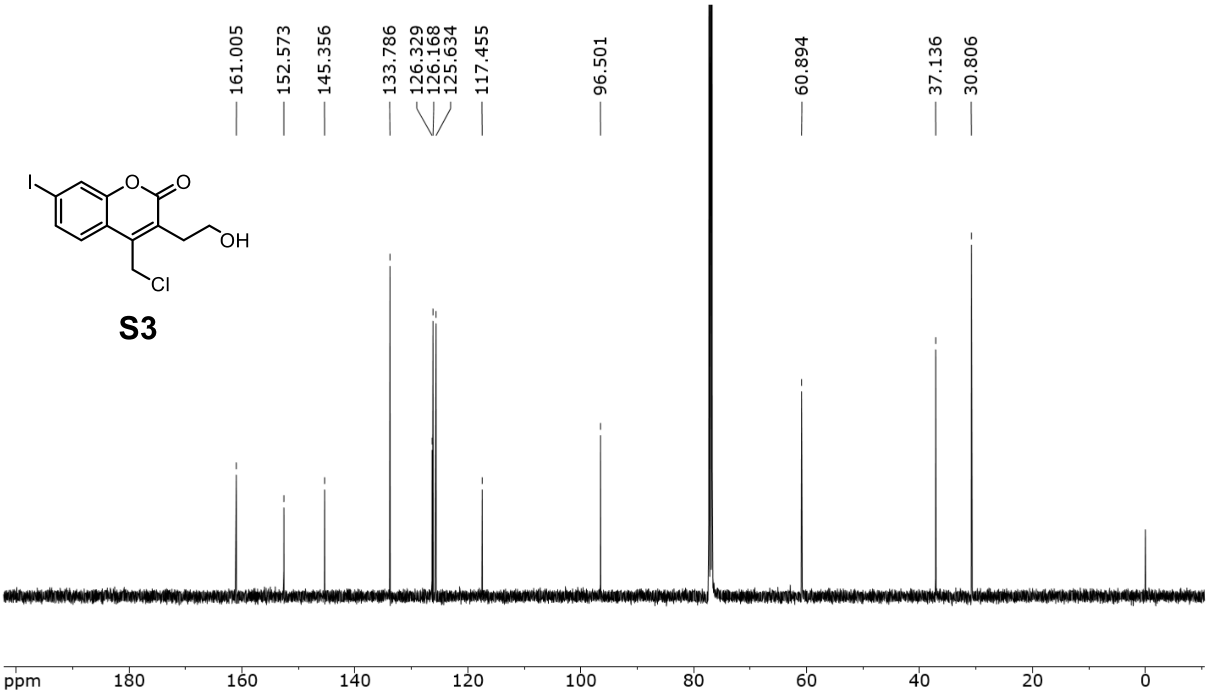

Fig. S18. $^{13}C\{^{1}H\}$ NMR spectrum of **S3** (126 MHz, $CDCl_3$, r.t.).

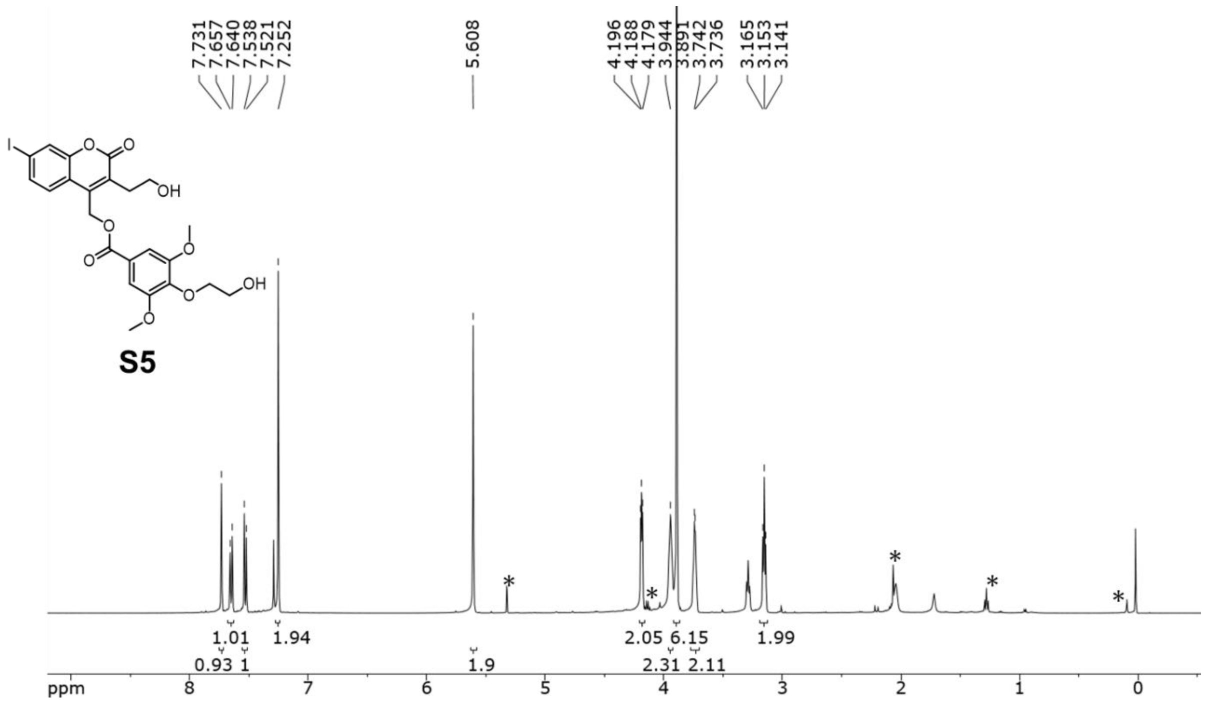

Fig. S19. ^{1}H NMR spectrum of **S5** (500 MHz, $CDCl_3$, r.t.). *Impurities: CH_2Cl_2, EtOAc, H grease, and silicon grease.

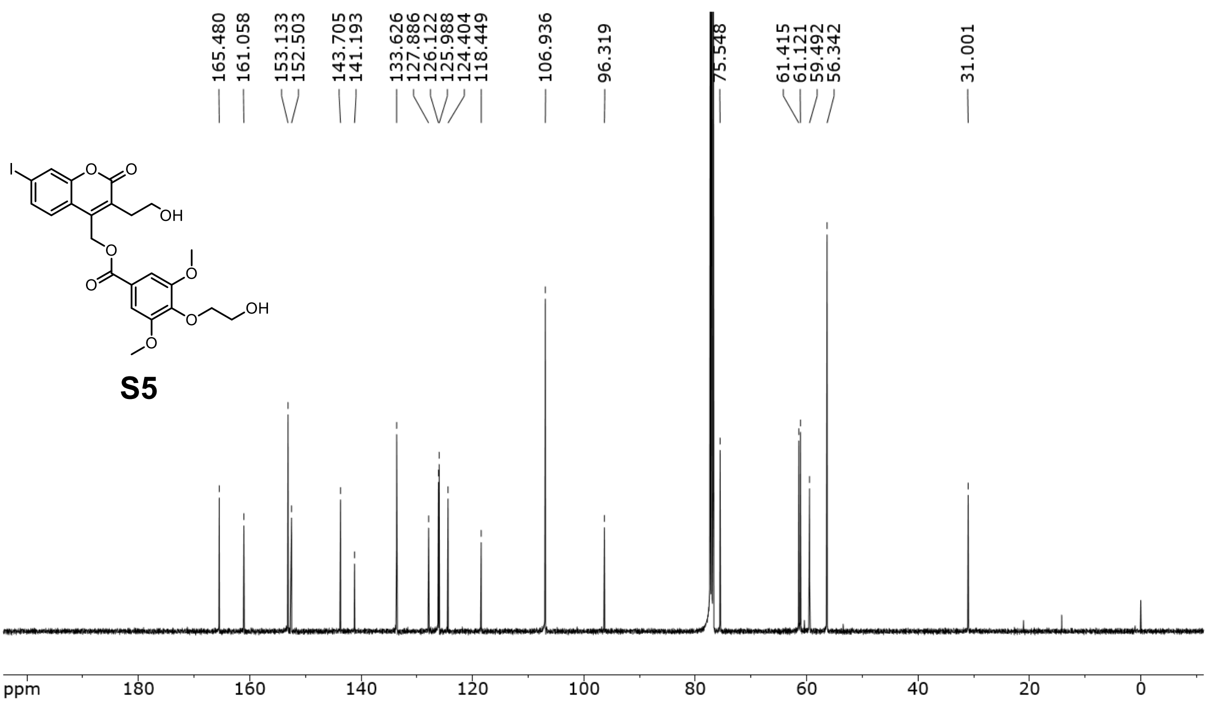

Fig. S20. $^{13}C\{^1H\}$ NMR spectrum of **S5** (126 MHz, $CDCl_3$, r.t.).

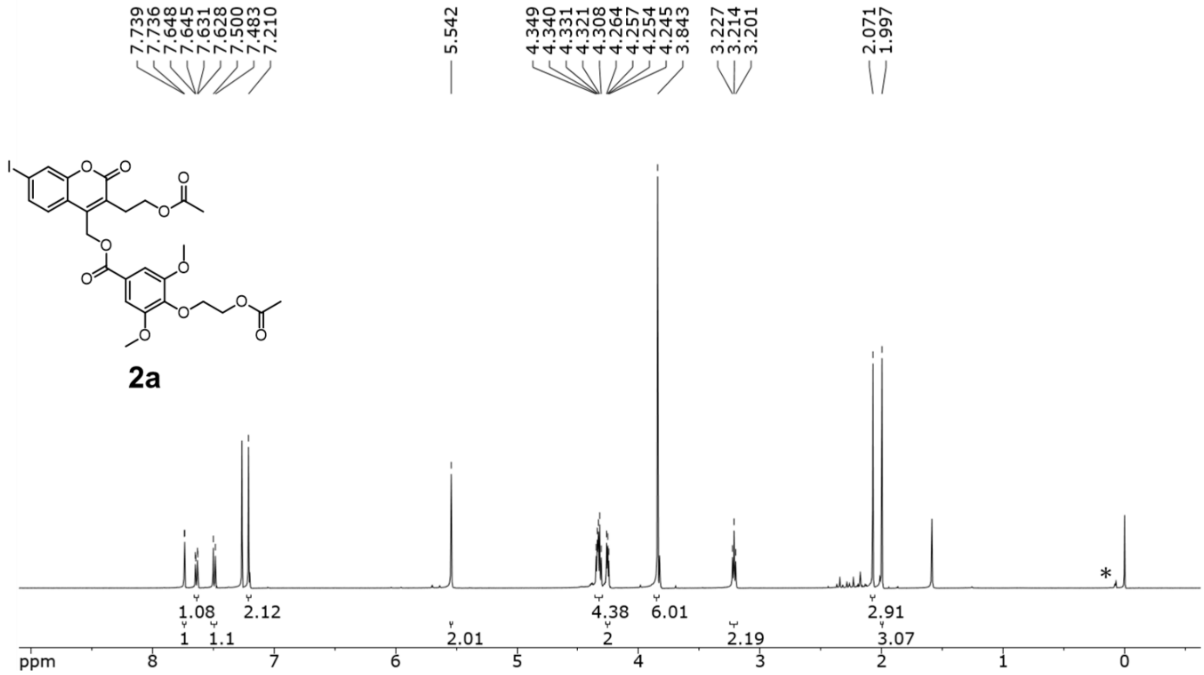

Fig. S21. ^{1}H NMR spectrum of **2a** (500 MHz, $CDCl_3$, r.t.). *Impurity: Silicon grease.

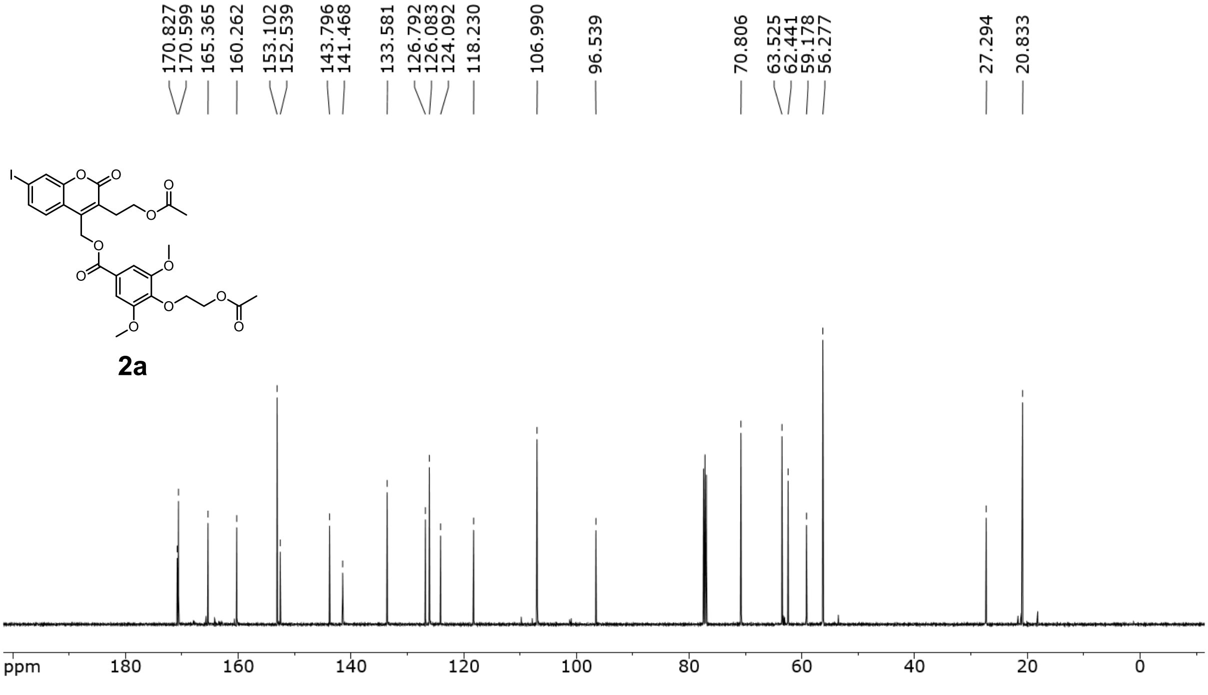

Fig. S22. $^{13}C\{^{1}H\}$ NMR spectrum of **2a** (126 MHz, $CDCl_3$, r.t.).

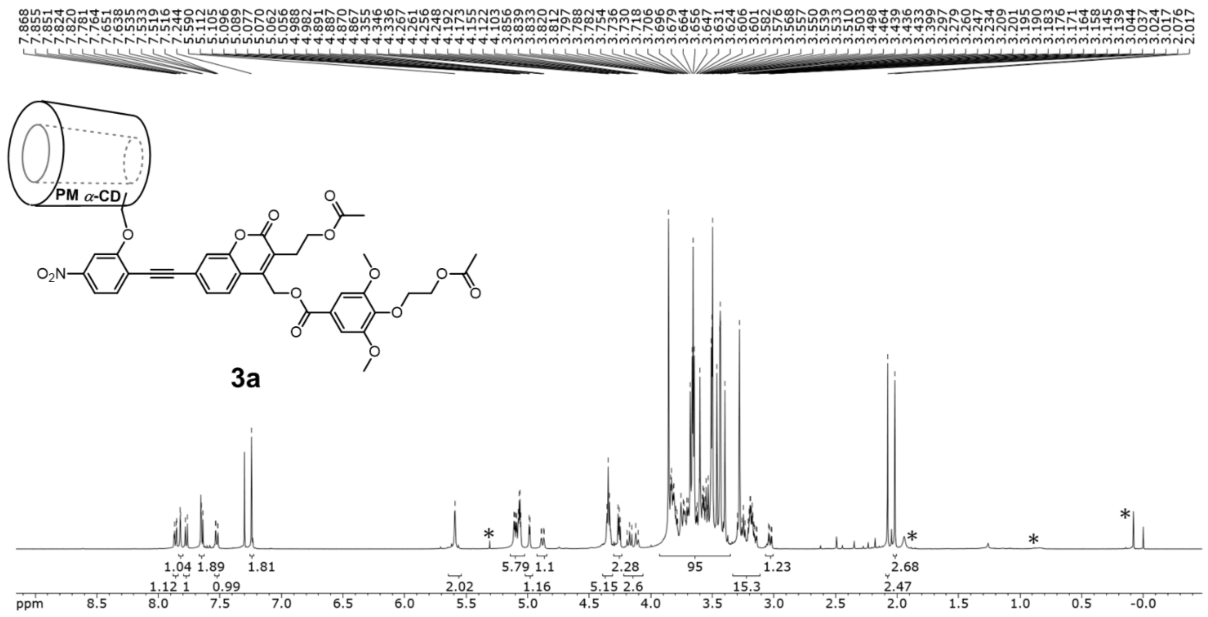

Fig. S23. ^{1}H NMR spectrum of **3a** (500 MHz, $CDCl_3$, r.t.). *Impurities: CH_2Cl_2, H grease, and silicon grease.

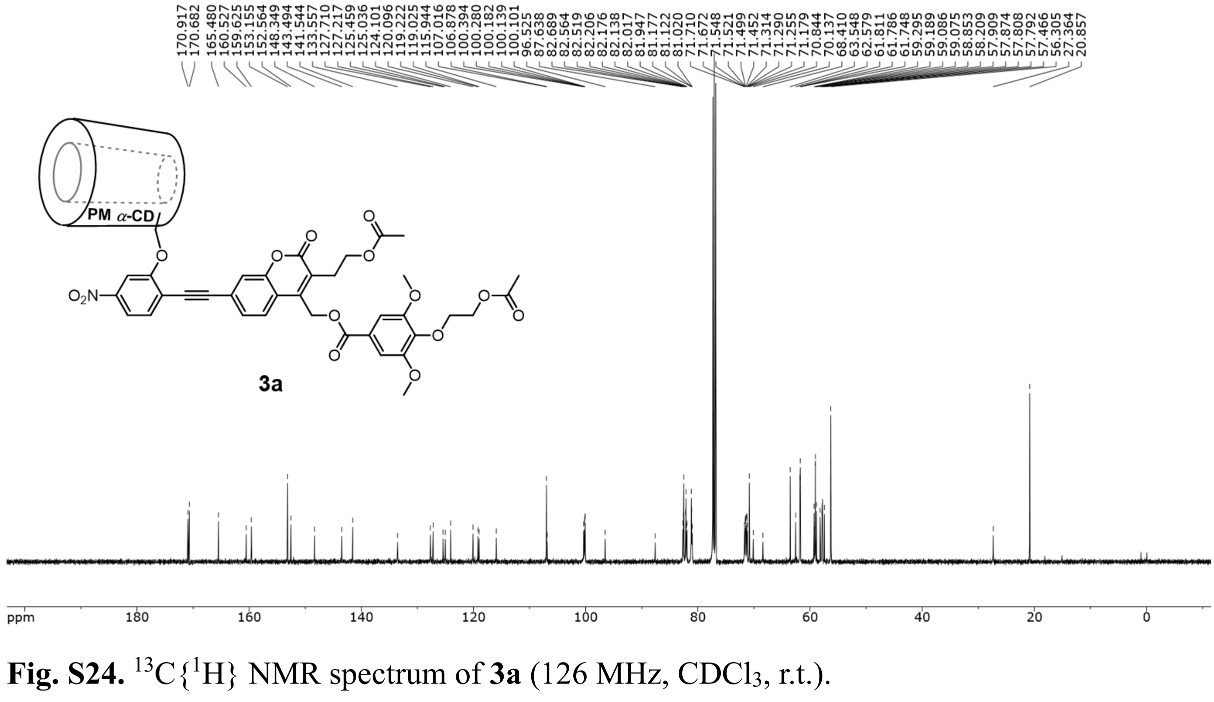

Fig. S24. $^{13}C\{^{1}H\}$ NMR spectrum of **3a** (126 MHz, $CDCl_3$, r.t.).

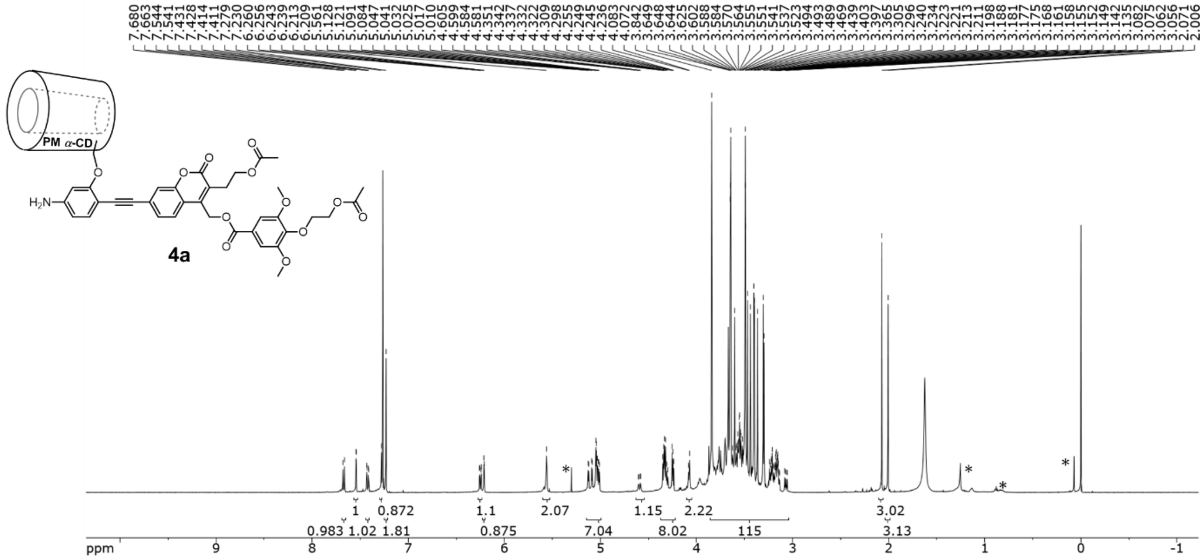

Fig. S25. ^{1}H NMR spectrum of **4a** (500 MHz, $CDCl_3$, r.t.). *Impurities: CH_2Cl_2, MeOH, H grease, and silicon grease.

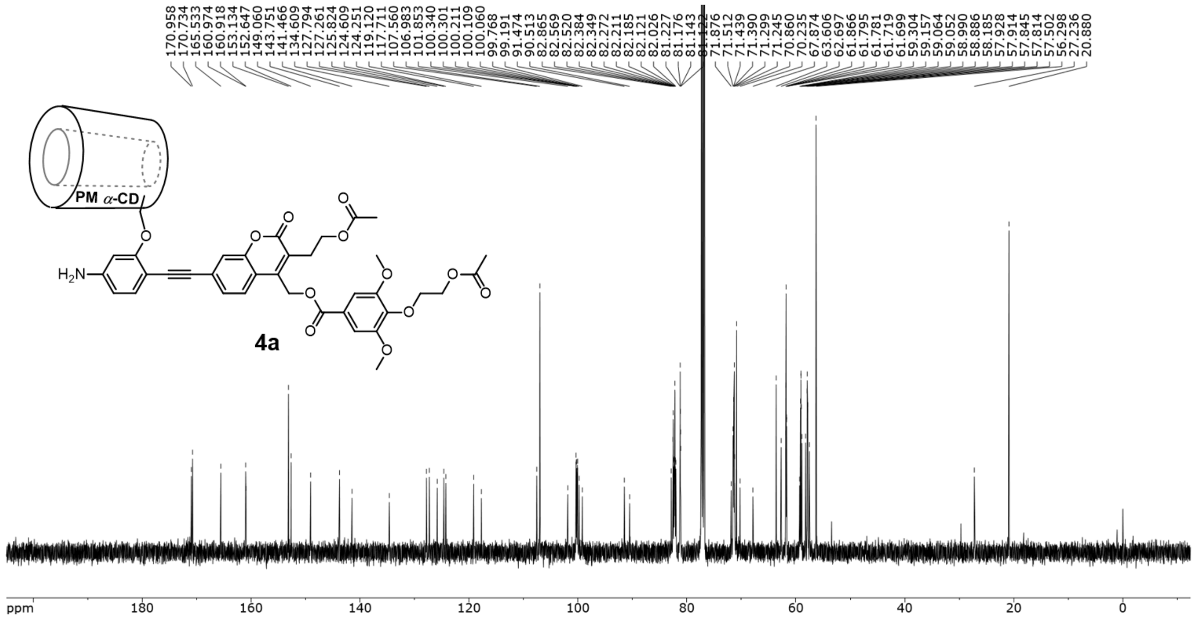

Fig. S26. ^{13}C{^{1}H} NMR spectrum of **4a** (126 MHz, $CDCl_3$, r.t.).

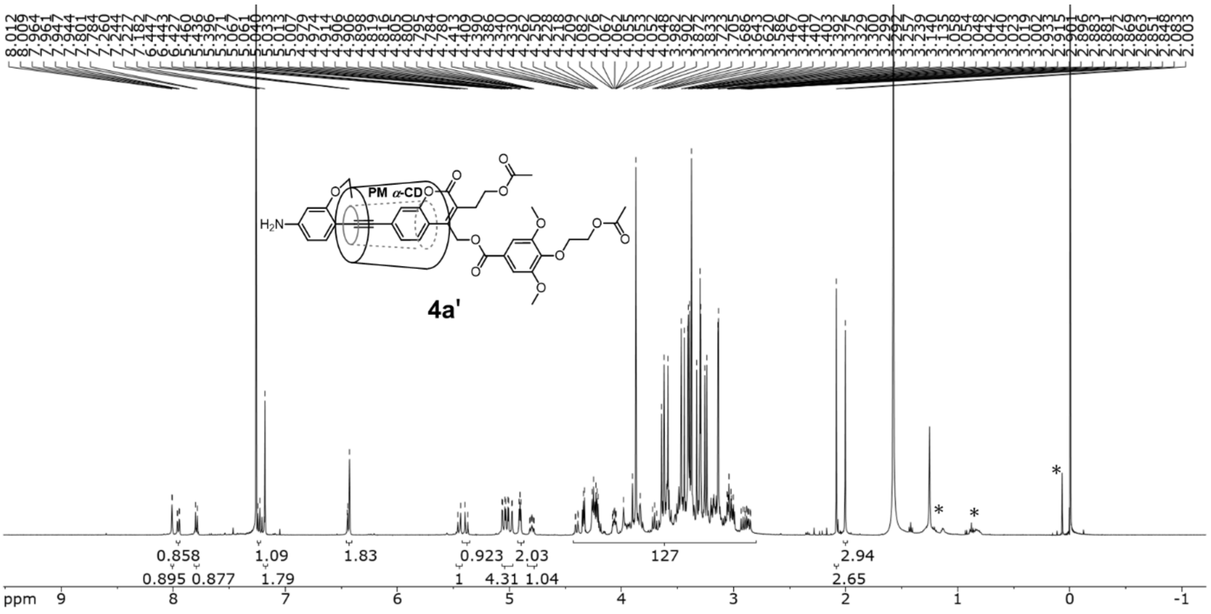

Fig. S27 ^{1}H NMR spectrum of **4a'** (500 MHz, $CDCl_3$, r.t.). *Impurities: H grease, and silicon grease.

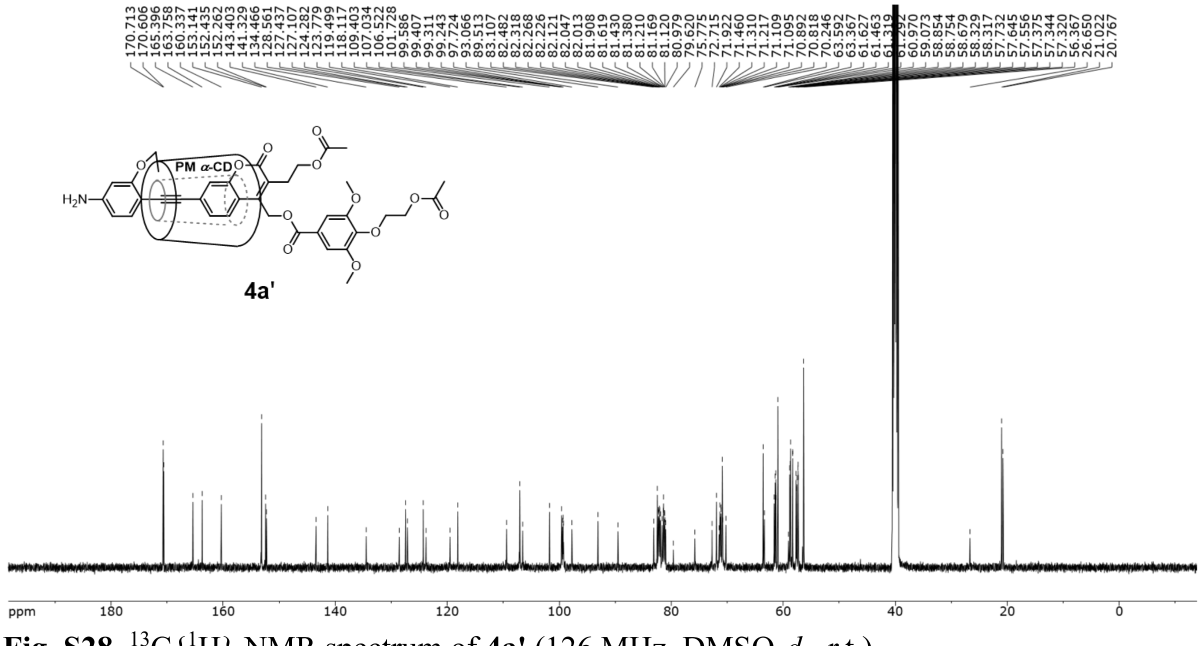

Fig. S28. $^{13}C\{^{1}H\}$ NMR spectrum of **4a'** (126 MHz, DMSO-d_6, r.t.).

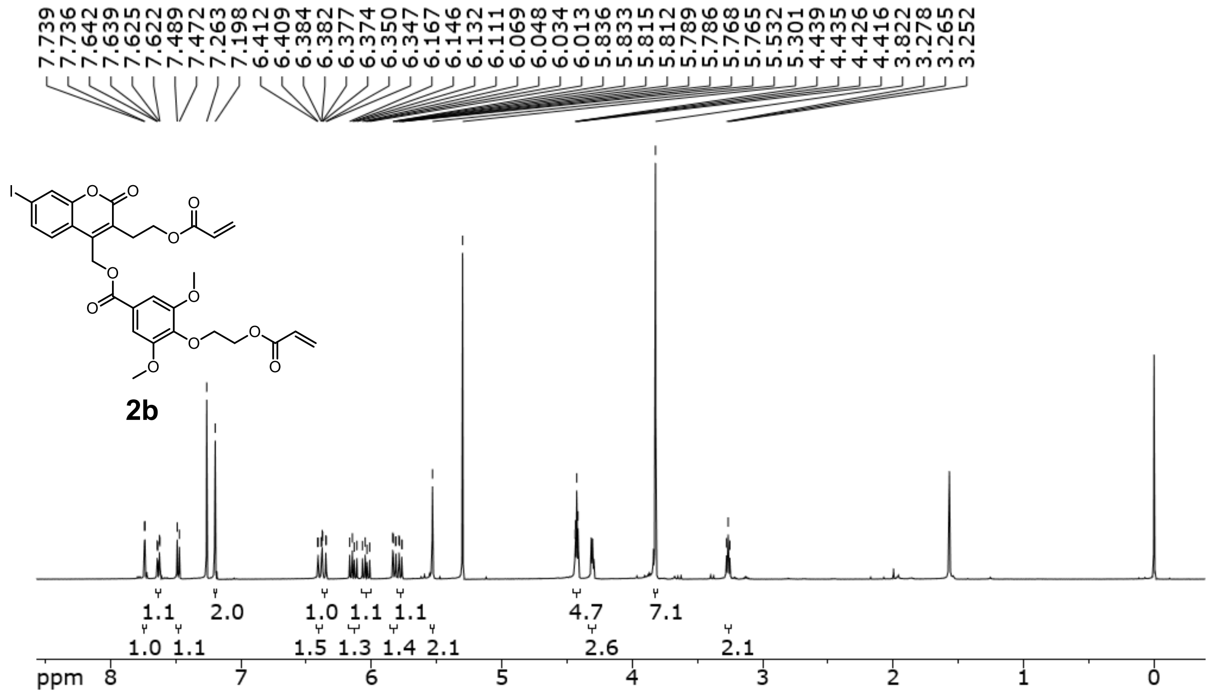

Fig. S29. ^{1}H NMR spectrum of **2b** (500 MHz, $CDCl_3$, r.t.).

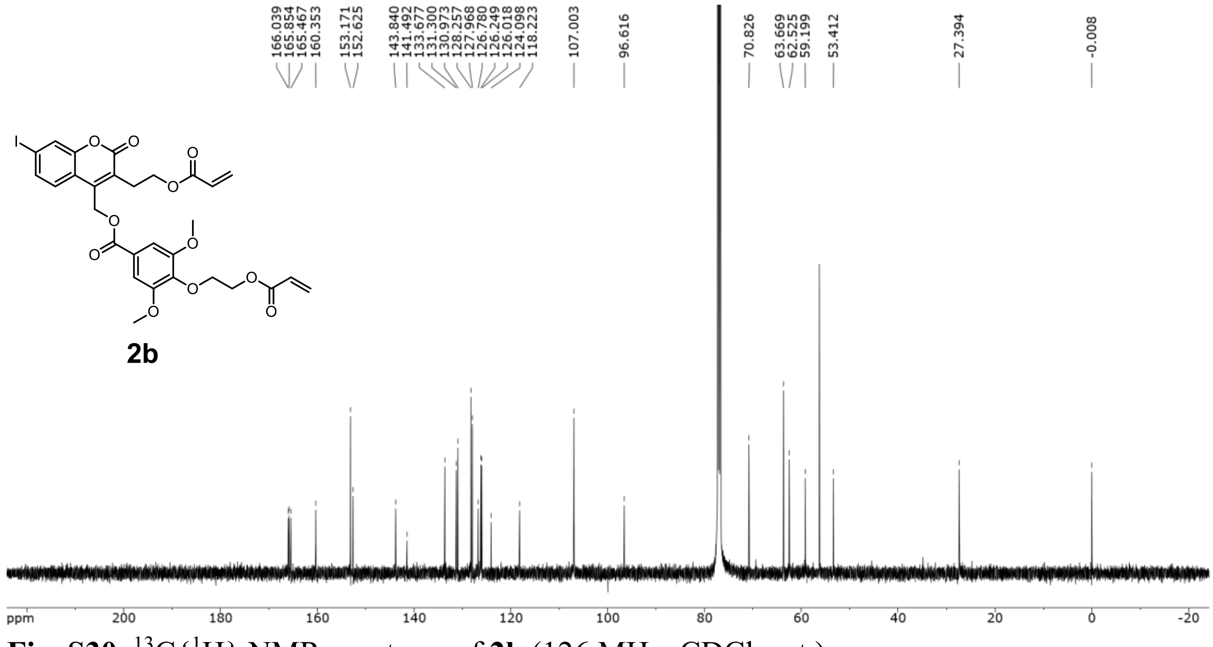

Fig. S30. $^{13}C\{^{1}H\}$ NMR spectrum of **2b** (126 MHz, $CDCl_3$, r.t.).

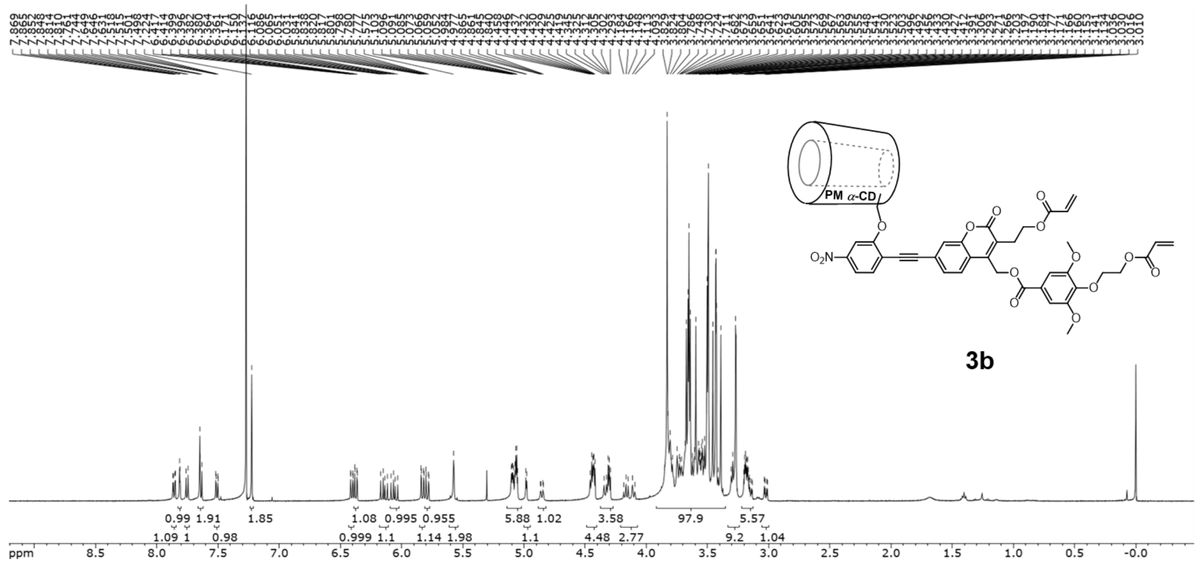

Fig. S31. ¹H NMR spectrum of **3b** (500 MHz, $CDCl_3$, r.t.).

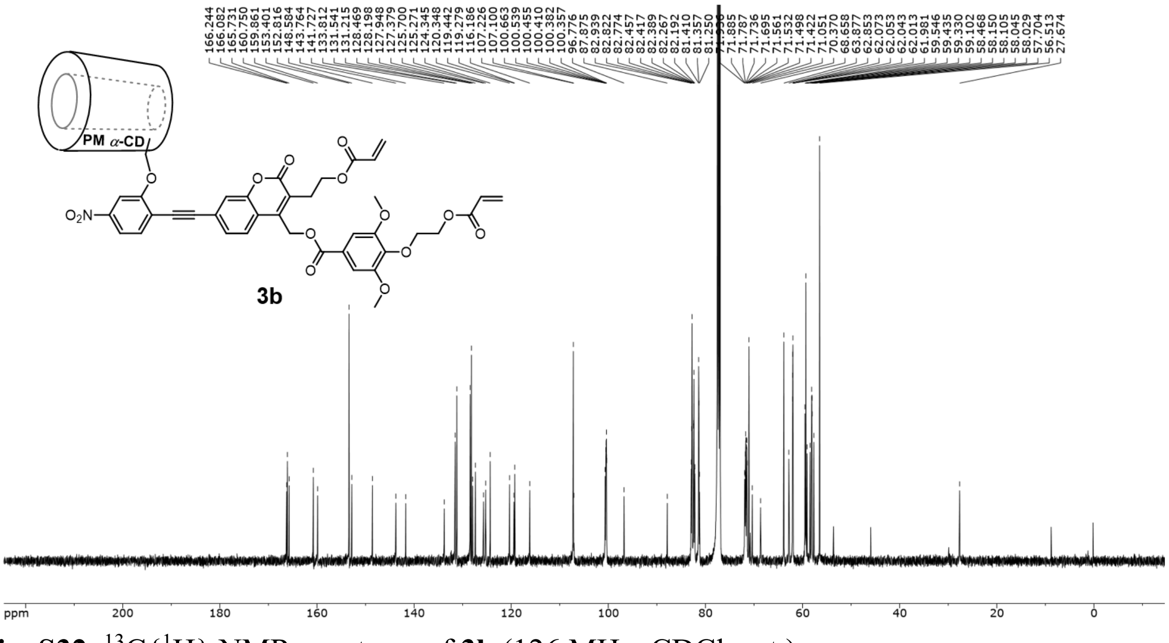

Fig. S32. ¹³C{¹H} NMR spectrum of **3b** (126 MHz, $CDCl_3$, r.t.).

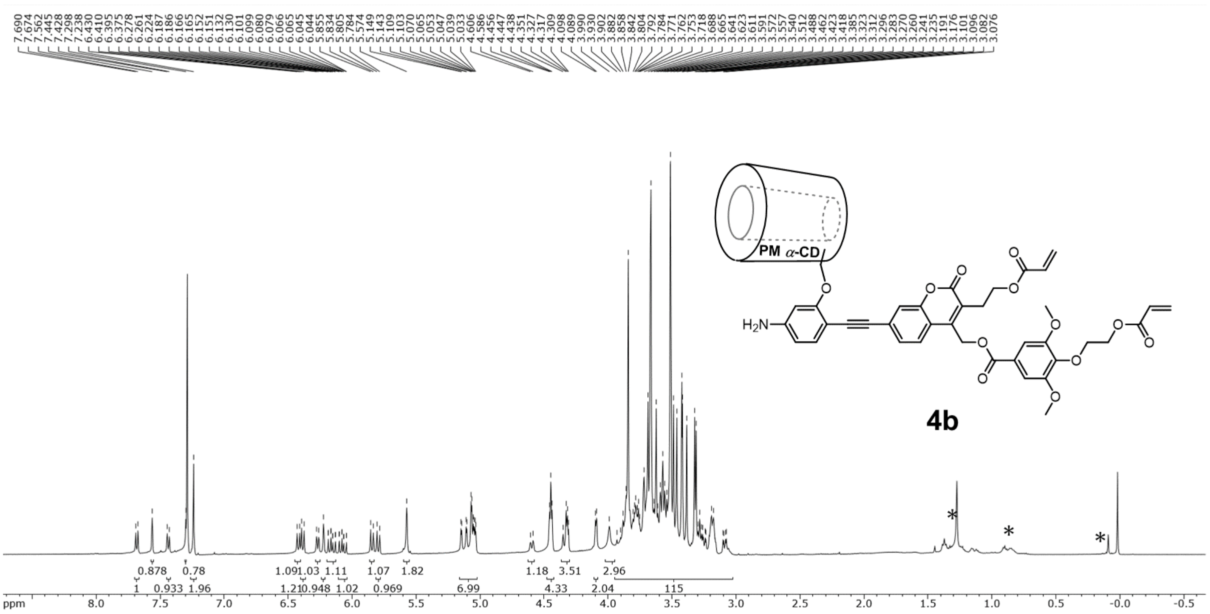

Fig. S33. ^{1}H NMR spectrum of **4b** (500 MHz, $CDCl_3$, r.t.). *Impurities: H grease, and silicon grease.

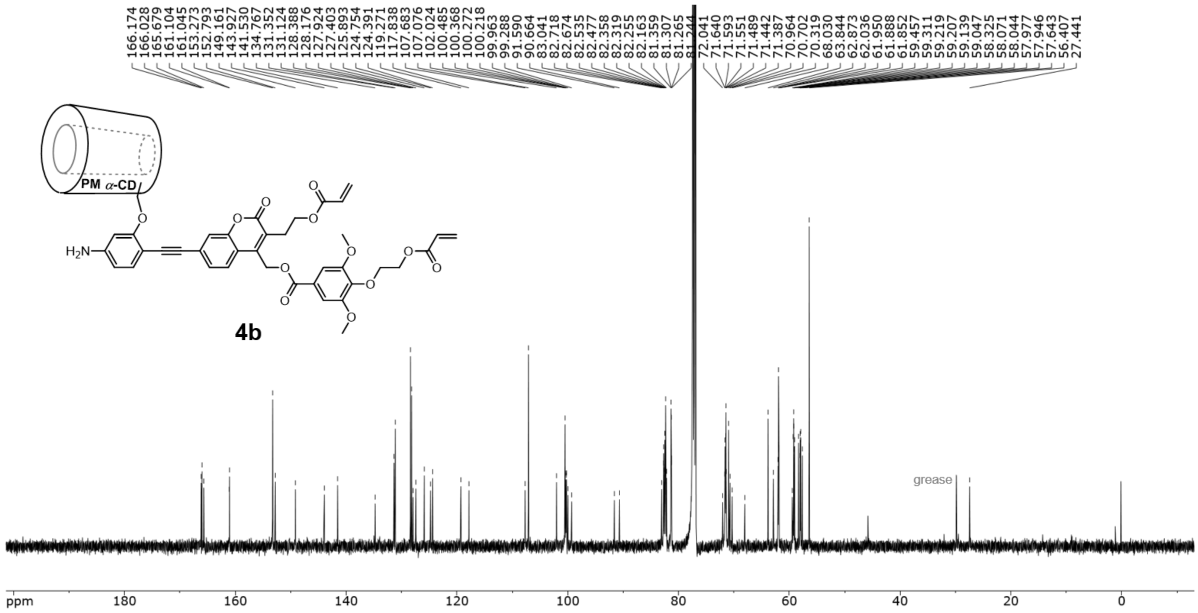

Fig. S34. $^{13}C\{^1H\}$ NMR spectrum of **4b** (126 MHz, $CDCl_3$, r.t.).

S12.2. ^1H-^1H ROESY NMR spectra

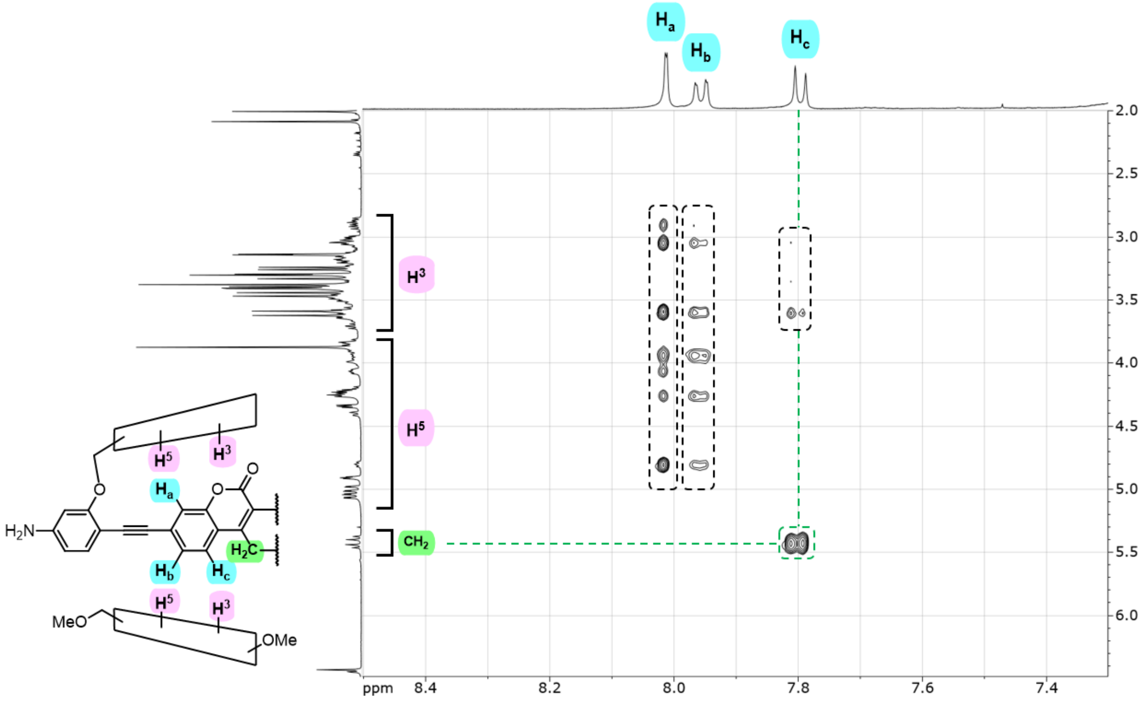

Fig. S35. ^1H-^1H ROESY NMR spectrum of **4a'** (500 MHz, $CDCl_3$, r.t.). A strong nuclear Overhauser effect was observed between π-system and inner protons on PM α-CD as evidence of the formation of the threading structure.

S13. References

1 H. Masai, J. Terao, S. Makuta, Y. Tachibana, T. Fujihara and Y. Tsuji, *J. Am. Chem. Soc.*, 2014, **136**, 14714–14717.

2 L. Mialon, R. Vanderhenst, A. G. Pemba, S. A. Miller, *Macromol. Rapid Commun.*, 2011, **32**, 1386–1392.

3 A. B. Pangborn, M. A. Giardello, R. H. Grubbs, R. K. Rosen and F. J. Timmer, *Organometallics*, 1996, **15**, 1518–1520.

4 M. J. Frisch, G. W. Trucks, H. B. Schlegel, G. E. Scuseria, M. A. Robb, J. R. Cheeseman, G. Scalmani, V. Barone, G. A. Petersson, H. Nakatsuji, X. Li, M. Caricato, A. V. Marenich, J. Bloino, B. G. Janesko, R. Gomperts, B. Mennucci, H. P. Hratchian, J. V. Ortiz, A. F. Izmaylov, J. L. Sonnenberg, D. Williams-Young, F. Ding, F. Lipparini, F. Egidi, J. Goings, B. Peng, A. Petrone, T. Henderson, D. Ranasinghe, V. G. Zakrzewski, J. Gao, N. Rega, G. Zheng, W. Liang, M. Hada, M. Ehara, K. Toyota, R. Fukuda, J. Hasegawa, M. Ishida, T. Nakajima, Y. Honda, O. Kitao, H. Nakai, T. Vreven, K. Throssell, J. A. Montgomery, Jr., J. E. Peralta, F. Ogliaro, M. J. Bearpark, J. J. Heyd, E. N. Brothers, K. N. Kudin, V. N. Staroverov, T. A. Keith, R. Kobayashi, J. Normand, K. Raghavachari, A. P. Rendell, J. C. Burant, S. S. Iyengar, J. Tomasi, M. Cossi, J. M. Millam, M. Klene, C. Adamo, R. Cammi, J. W. Ochterski, R. L. Martin, K. Morokuma, O. Farkas, J. B. Foresman, and D. J. Fox, Gaussian 16, Revision C.02, Gaussian, Inc., Wallingford CT, 2019.
